# There is plenty of room at the top: generation of hot charge carriers and their applications in perovskite and other semiconductor-based optoelectronic devices

**DOI:** 10.1038/s41377-021-00609-3

**Published:** 2021-09-01

**Authors:** Irfan Ahmed, Lei Shi, Hannu Pasanen, Paola Vivo, Partha Maity, Mohammad Hatamvand, Yiqiang Zhan

**Affiliations:** 1 State Key Laboratory of ASIC and System, Centre of Micro-Nano System, SIST, Fudan University, 200433 Shanghai, China; 2 Department of Physics, Government Postgraduate College, (Higher Education Department-HED) Khyber Pakhtunkhwa, 21300, Mansehra, Pakistan; 3 State Key Laboratory of Surface Physics, Key Laboratory of Micro- and Nano-Photonics, Fudan University, 200433 Shanghai, China; 4 Faculty of Engineering and Natural Sciences, Tampere University, FI-33014 Tampere, Finland; 5 KAUST Solar Center, Division of Physical Science and Engineering, King Abdullah University of Science and Technology (KAUST), Thuwal, 23955-6900 Riyadh, Kingdom of Saudi Arabia

**Keywords:** Electronics, photonics and device physics, Optical materials and structures

## Abstract

Hot charge carriers (HC) are photoexcited electrons and holes that exist in nonequilibrium high-energy states of photoactive materials. Prolonged cooling time and rapid extraction are the current challenges for the development of future innovative HC-based optoelectronic devices, such as HC solar cells (HCSCs), hot energy transistors (HETs), HC photocatalytic reactors, and lasing devices. Based on a thorough analysis of the basic mechanisms of HC generation, thermalization, and cooling dynamics, this review outlines the various possible strategies to delay the HC cooling as well as to speed up their extraction. Various materials with slow cooling behavior, including perovskites and other semiconductors, are thoroughly presented. In addition, the opportunities for the generation of plasmon-induced HC through surface plasmon resonance and their technological applications in hybrid nanostructures are discussed in detail. By judiciously designing the plasmonic nanostructures, the light coupling into the photoactive layer and its optical absorption can be greatly enhanced as well as the successful conversion of incident photons to HC with tunable energies can also be realized. Finally, the future outlook of HC in optoelectronics is highlighted which will provide great insight to the research community.

## Abstract (web summary)

In photoactive materials, the fundamental understandings of hot charge carriers and a successful device design are the current challenges for the development of highly efficient hot carrier optoelectronic devices.

## Graphical abstract

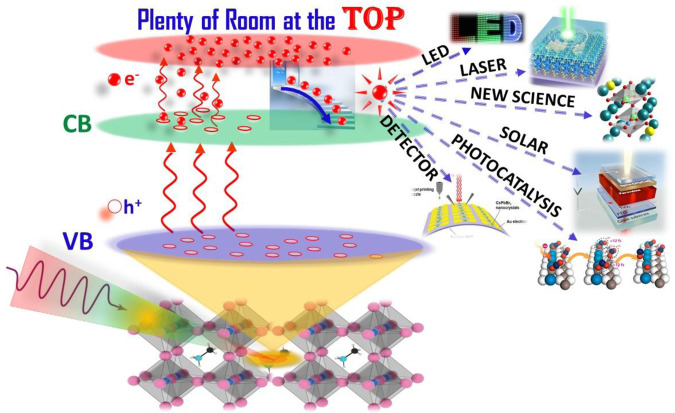

## Introduction

**Fig. 1:**
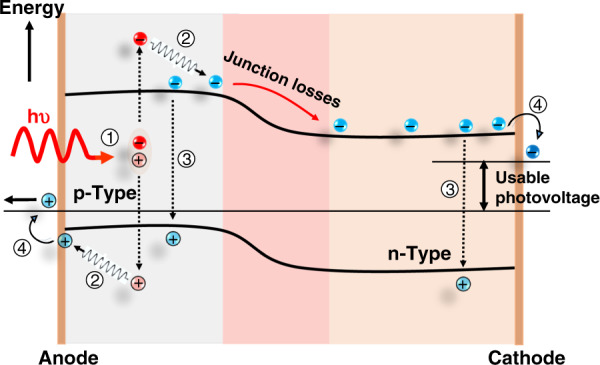
Pathways of primary energy loss in a conventional single-junction solar cell: (1) photoexcitation (2) hot charge carrier loss, (3) recombination losses, and (4) contact losses. The figure is taken from ref. ^32^ and modified

The quest for a sustainable society has promoted rapid research progress and stride in the field of optoelectronics. With a skyrocketing increase in the energy demand, there is an urgent need for a highly efficient, cost-effective, and environmentally stable energy technology to foster the rapid development of emerging scenarios like nearly zero-energy buildings, solar vehicles, portable electronic devices, ultrafast lasers, sensors, light-emitting diodes (LEDs), and photocatalytic devices. Several factors, such as production cost, price of precursor materials, and their toxicity, need to be carefully considered by the respective stakeholders. However, power conversion efficiency (PCE) is the key criterion that has been typically tackled by the scientific community in the last few decades. In 1961, Shockley and Queisser demonstrated a theoretical PCE limit (33.8%) in solar cells^1^. This limit has been already surpassed with the effective employment of the multijunction concept, whereby multiple semiconductor layers, with bandgaps tuned to broaden the coverage of the solar spectrum, are combined in the same device^2,3^. The energy of the solar photons lies in the range between 0.5 and 3.5 eV. The photons with energy below the bandgap of the photoactive material are not absorbed while those with energy above the bandgap, create electron and hole (e–h) pairs with excess kinetic energy equal to the difference between the incident photon energy and the bandgap energy of the material^4,5^. These (e–h) pairs are referred to as hot charge carriers (HC). In conventional solar cells, the excess energy of photons over the bandgap is wasted as heat. The general description of the solar cells working mechanism, charge collection, and energy loss is presented in Fig. 1^6^.

The effective utilization of such excess energy can be achieved by two alternative processes, namely (i) enhancing the photo-voltage by extracting the HC before their quick cooling, or (ii) increasing the photocurrent by producing one or two more e–h pairs through impact ionization^7^. The first process refers to the hot-carrier solar cells (HCSCs)^8^ and the second one is known as multiple exciton generation (MEG) or carrier multiplication (see the section “HC cooling and multiple exciton generation (MEG)”)^9^. HC extraction is an unconventional and innovative approach to overcome the unavoidable energy losses in solar cells. As a result, HC enable boosting the PCE towards the Shockley–Queisser (S–Q) limit and beyond. The concept is based on the thermal isolation of HC and phonons (so that they may stay at different temperature regimes^10^), followed by their selective extraction to the external circuit through efficient energy-selective contacts (ESCs).

Considering the theoretical S–Q limit of single-junction solar cells (under 1 and 100 Sun illuminations), there is still plenty of room for sophisticated device architectures and new materials. Since the first theoretical concept introduced by Ross and Nozik in 1982 on HC solar energy converters, many researchers across the globe struggled with developing a proof-of-concept device architecture^4^. The collection of excited charges in femto- to picosecond time regime, before their subsequent cooling toward the respective conduction band, is a daunting challenge for the practical application of this concept into a fully functional device^11^.

HC are soaked in a fluid of phonons and at first glance, it is very difficult to isolate them from such a highly unstable fluid. In his book about the energy losses by the charge carriers in solar cells, Ridley highlights that “*Interest in semiconductor physics generally focuses on properties consequent on the quantum transitions made by electrons and holes, but many of these transitions involve the emission of phonons, and therefore it is pertinent to ask what happens to these phonons*”^12^. The strong interaction of HC with acoustic and optical phonons, the scattering of these phonons from the nanostructure and boundaries, and their contribution to the thermal conductivity are important processes that need to be addressed.

**Fig. 2:**
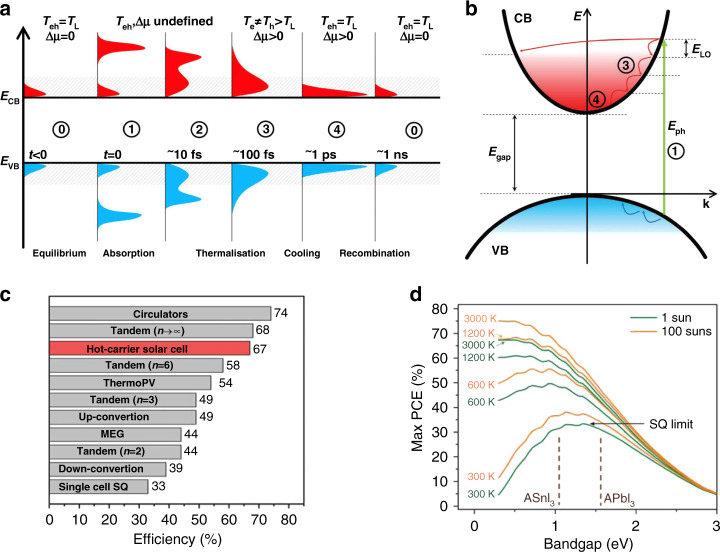
The schematics represent an arbitrary narrow bandgap semiconductor with the distribution of charge carriers in the two energy bands (VB and CB) and efficiencies of various new generation solar cells which crosses the S-Q limit. **a** The sequential process of HC, starting from excitation till recombination. **b** The cooling of HC in their respective bands of unequal curvatures (as different carrier effective mass). **c** The new generation solar cells with efficiency above the S–Q limit. **d** Under irradiation of 1 and 100 suns concentration (i.e., low-concentrator photovoltaics) the ultimate efficiency of HC solar cells, at different charge carrier temperatures, plotted versus their photoabsorber bandgap. A conventional solar cell is represented by the lowermost curve, while the vertical dashed lines correspond to the bandgap of halide perovskite materials. “A” in ASnI_3_ and APbI_3_ corresponds to methylammonium (MA), formamidinium (FA), and cesium (Cs) ions. The figures are adopted with permission from (**a**, **b**) ref. ^14^ and (**c**, **d**) ref. ^26^

The schematics of photoexcitation, along with HC generation and the sequential process of their thermalization and recombination, are illustrated in Fig. 2a. The cooling of HC in their respective conduction and valence energy bands (hot electrons in CB and hot holes in VB, respectively) takes place through the generation of phonons (Fig. 2b)^13^. The PCE of HC can be extended up to 67%, close to that of tandem cells at 1 Sun irradiation (Fig. 2c), and can even reach up to ≈75% using 100 sun concentrators, where the electronic temperature increases up to 3000 K (Fig. 2d). This highlights the importance of this new type of device (HCSCs).

Compared to multijunction solar cells that require various stacked layers of different semiconductors, HCSC is composed of only three layers, i.e., an absorber and two ESC layers (for extraction of hot electrons and hot holes, respectively)^14,15^. This simple architecture can achieve similar PCE as that of multijunction photovoltaics (PV). Studies on HC have been also extended to various other important applications beyond PV, such as photocatalysis^16^, photodetection^17^, and light emission^18^.

The advent of a new class of photoactive materials with outstanding optoelectronic properties, namely halide perovskites, has created exciting research avenues uncovering new technological applications. Perovskite materials have been extensively studied in the context of photon absorption and separation/collection of charge carriers, leaving the HC generation, thermalization, and extraction aspects almost unexplored.

On the other hand, metallic nanostructures (typically Ag, Au, and Cu) can also produce HC. When the frequency of incident light matches the intrinsic oscillation of free electrons in the metallic nanostructure, the electrons oscillate collectively, which is known as surface plasmon resonance (SPR)^19,20^. The generation of HC is one of the energy-releasing mechanisms of these plasmons as they undergo ultrafast dephasing in femto- to picosecond timescale. The fundamental understanding of excitation, generation, and successful extraction of these plasmonic HC as well as their futuristic use in photodetection, sensing, photochemical, and PV applications are the hot research topic these days. Deepening the knowledge on higher excited energy states, as well as HC, will not only enable the development of sophisticated ultrafast spectroscopy techniques but will also open new research directions for a number of energy conversion applications based on enhanced light intensities, hence making the realization of future HC optoelectronics possible.

This review addresses the fundamental concept of HC generation in photoactive materials, with a special focus on their thermalization and cooling phenomenon (see section “HC generation, thermalization, and relaxation phenomena”). Section “HC in organic–inorganic halide perovskites” highlights the key properties of HC in perovskite materials followed by the effect of material morphology and structure on the HC behavior. The design and performance of efficient energy-selective contacts for perovskites and other semiconductor materials are discussed in the section “Working principals of HC solar cells”. Furthermore, the basic plasmonic effects in the most common plasmonic materials, as well as their energy loss mechanisms in terms of HC generation, are elucidated in the section “Plasmonic HC and their applications in other optoelectronic devices”, which also show their effective use in futuristic energy-efficient applications, such as ultrafast photochemical reactors, hot-electron transistors (HET), detectors, and lasing devices. Finally, in the section “Outlook for HC in future optoelectronic devices”, we highlight the most promising research perspectives and directions for HC science and applications.

## HC generation, thermalization, and relaxation phenomena

**Fig. 3:**
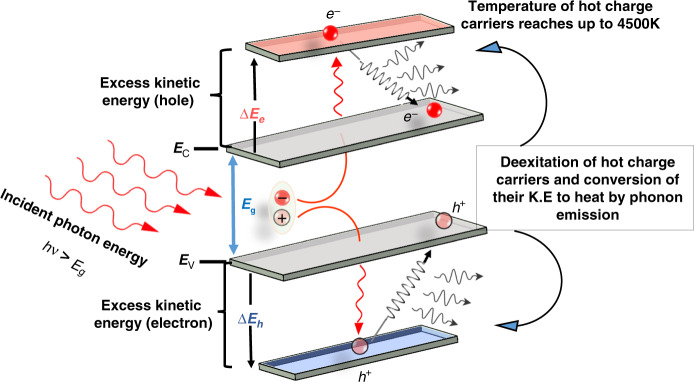
Absorption of an incident photon carrying energy higher than the bandgap energy (*E*_g_) of the photoabsorber. The excitation of an electron leaves behind a positive charge (hole). Excited electrons and holes are collectively called HC, whose excess energy is Δ*E*_*e*_ and Δ*E*_*h*_ for hot electrons and hot holes, respectively. Upon de-excitation, the excess energy is converted to heat by the emission of phonons

**Fig. 4:**
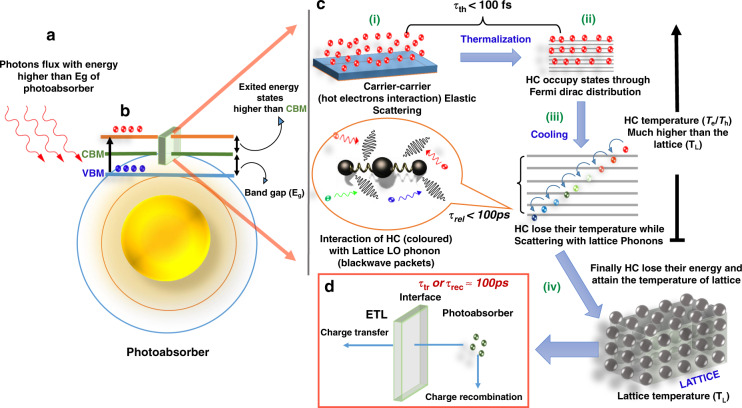
The formation of HC, their interactions, and finally the cooling down to lattice temperature. In this image, “HC” refers to hot electrons. A similar phenomenon takes place for hot holes as well. **a** Absorption of high-energy photons by a photoabsorber. **b** Electrons from the valence band maximum (VBM) of an absorber material jump to energy states higher than conduction band minimum (CBM). The detailed phenomenon of HC is presented in (**c**): (i) carrier–carrier scattering, (ii) the thermalization of HC to occupy energy states through Fermi–Dirac distribution, (iii) cooling through their interaction with LO phonons, and (iv) finally the loss of their energy to attain lattice temperature. **d** The charge carrier either transfer to the electron-transport layer (ETL) or recombination takes place

In the photoexcitation process, an incident photon, whose energy is equal to or higher than the bandgap energy (*E*_g_) of the targeted materials, imparts its energy to the valence band electron of the photoabsorber material and excites it to the higher energy level (conduction band). The excited electrons in the conduction band leave a positive charge behind in the valance band, termed as an excited hole. These excited species are collectively called “excitons” or “free charge carriers”. However, if the excitation energy is far higher than *E*_g_, the charge carriers jump to even higher energy states, termed as energy sub-bands, which are above the conduction band minimum (CBM) and below the valence band maximum (VBM) for excited electrons and excited holes, respectively. The highly excited charge carriers are hot electrons and hot holes, collectively termed as HC, as shown in Fig. 4 (see ref. ^21^). HC convert their excess energy to heat upon de-excitation. The distribution of the excess energy between the HC is described by the following equations^22^:

1 $${\Delta}E_e =({h\nu - E_g})\bigg[{1 + m_e^ \ast \big/\!_{{m_h}^ \ast}} \bigg]^{-1}$$

2 $${\Delta}E_h = \left( {h\nu - E_g} \right) - {\Delta}E_e$$

where m^*^_e_ and m^*^_h_ are the effective masses of electron and hole, respectively. Δ*E*_*e*_ is the energy difference between the conduction band and the initial energy of photogenerated electrons, and Δ*E*_*h*_ is the energy difference between the valence band and photogenerated holes (see Fig. 3, Eqs. (1) and (2)).

The HC, generated through photoexcitation, subsequently cool down by carrier–carrier collision, scattering between HC and lattice phonons, causing significant energy losses (thermal cooling). The general mechanism of HC generation and cooling is summarized in Fig. 3. The scattering continues until the energy of HC is less than the longitudinal optical (LO) phonon energy. The emitted LO phonons from the electron–LO–phonon scattering decay into daughter acoustic phonons or transverse optical phonons, which decay further within 100 ps. The thermally cooled HC are now available to be transferred to the charge transport layers (hole-transport layer, HTL, or electron-transport layer, ETL) or lose the remaining part of their energy through recombination. The detailed mechanism of HC generation and their subsequent thermalization and cooling processes are illustrated in Fig. 4a–d.

The HC relaxation process is composed of two stages. In the first stage, the HC are far from the equilibrium, and the collisions start between the HC (electron–electron and hole–hole collision) or through impact ionization and Auger recombination if their concentration is high (>10^18^ cm^−3^)^23,24^. This stage is attained very rapidly (<100 fs) and is referred to as “thermal equilibration” or “carrier thermalization”^22^. The temperature of these species generally reaches from 2500 to 4500 K under 1 and 100 sun illumination, respectively^22,25^. The second stage of HC relaxation starts with their equilibration with the lattice through carrier–phonon inelastic interaction until the temperature of both lattice and HC becomes equal. The process is referred to as “carrier-cooling” and occurs at the picosecond timescale. Finally, the equilibration ends with the complete relaxation of the system. Electrons and holes are now available in their respective energy bands for either transfer to the charge transport layers (in the case of PV devices) or for recombining through radiative or nonradiative processes.

Two important clarifications need to be made before discussing the HC and the related energy dissipation phenomena in more detail: (i) for the calculation of the energy loss rate per charge carrier, the ultrafast community has standardized and fixed the minimum value of HC temperature to 600 K. In fact, below 600 K the cooling rate of HC is sufficiently slow, which may underestimate the results; (ii) due care must be considered when reporting the values of HC lifetime because every spectroscopic instrument, such as those required to carry out the time-resolved photoluminescence (TRPL) or transient absorption (TA) characterization, has its own temporal response that may influence the value of HC intrinsic lifetime. The detailed processes of HC relaxation are elaborated in the following sub-sections.

### Pathways during thermalization and cooling of HC

As explained by Li et al., in higher excited energy states, the HC redistribute their energy and relax through various pathways to attain the thermal equilibrium with the lattice^26^. The energy relaxation process is explained by the concepts of “carrier thermalization” and “carrier cooling”.

The carrier thermalization occurs very rapidly (<100 fs) and is governed by carrier–carrier elastic collisions in which the HC equilibrate among themselves. However, when the HC concentration is high due to the high excitation density of the incident light, an equilibration process also occurs through an impact ionization and Auger recombination^5,24^. The HC scattering is generally proportional to the square of their concentration (electrons or holes)^27^ that leads to the renormalization of HC or it interacts with the valence band electrons (in the case of hot electrons) known as the Auger recombination. Auger recombination is not a loss mechanism but rather a reverse process and, once the energy is released, the free charge carriers are re-heated again^5^. The energy renormalization process results in a Fermi–Dirac distribution of the hot species that reaches a higher temperature than their lattice^5^. These hot species interact amongst themselves by carrier–carrier interactions and intervalley scattering to achieve a Fermi–Dirac energy distribution (separately for hot electrons and hot holes)^14^. A separate temperature is assigned to hot electrons and hot holes, which reflects the distribution of kinetic energy in their respective charge carrier population. The equilibrated HC occupy energy states according to the Fermi–Dirac statistics with a temperature assigned as carrier temperature, *T*c (*T*_e_ and *T*_h_ for electrons and holes, respectively) that is larger than the lattice temperature (*T*_L_)^28^. This distribution is maintained by the carrier–carrier interactions to randomize the carrier distribution in *k*-space, where the *k*-space randomization is affected by the interaction rate. An interested reader may refer to refs. ^25,29^ for further detailed insights. Although, the hot species attain an equilibrium among themselves, they are far from equilibrium when compared to the lattice, and no phonon generation has yet taken place at this stage.

The carrier-cooling stage, taking place after carrier thermalization, starts with the equilibration of thermalized carriers with the crystalline lattice mainly through inelastic carrier–phonon interactions.^5^ The excess kinetic energy is transferred from the carriers to the phonons, in which the carrier cooling and the lattice heating take place until both reach the thermal equilibrium^22^.

#### Mechanisms for optical phonon emission

**Fig. 5:**
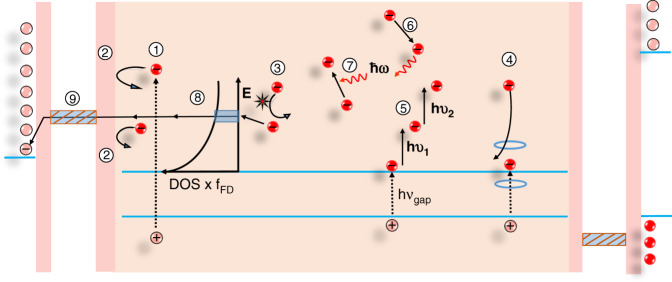
The energy loss mechanisms of HC (hot electrons only), and their transfer to energy-selective contacts (ESCs). The numerical labels indicate each specific process, namely (1) exciton generation, (2) hot electrons reflection at the ESC, (3) electron–electron collision, (4) impact ionization, (5) sub-bandgap photons reabsorbed by free electrons, (6) emission of optical phonon, (7) re-absorption of optical phonon, (8) quasi-ballistic transport of HC, (9) tunneling of HC from the photoabsorber to the interfacial contact. The figure is taken with permission from ref. ^5^

Because of the local electrostatic distortion, the Fröhlich interaction starts between the electrons and phonons and results in the formation of polarons^5^. Furthermore, the electron energy loss takes place through the emission of an optical phonon, which decays further to an acoustic phonon that reheats the free electrons^5^. The generalized process of HC generation and the possible scattering of HC are depicted in Fig. 5.

This process continues with phonon emission as long as the *T*_*C*_ of HC is higher than that of the phonons^30^. *T*_C_ is extracted from the high-energy tail of the TA spectra using the Maxwell Boltzmann function (Eq. (3)). In halide perovskites, the effective masses of an electron and hole are roughly the same (m^*^_e_ = 0.19 m_0_, m^*^_h_ = 0.25 m_0_, where m_0_ is the rest mass of an electron)^24,26,31^, and thus the temperature of hot electrons and hot holes are approximately equal^26^.

3 $${\Delta}A = - A_0\left( E \right)\exp \left( { - \frac{{{\Delta}E}}{{K_BT_C}}} \right)$$

where, Δ*A* is a transient absorption, ${\Delta}E\,(E_f - E)$ i.e., *E*–*E*_f_ > *K*_*B*_*T*, *T*_C_ is the HC temperature and *K*_*B*_ is the Boltzmann constant.

#### Mechanism of phonon decay

**Fig. 6:**
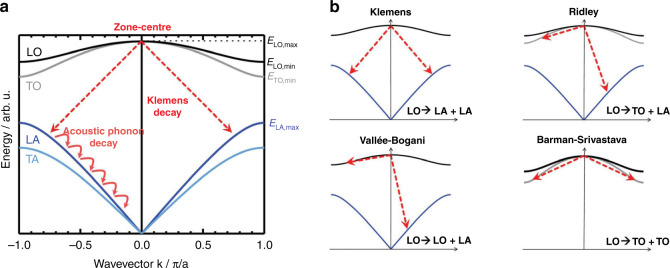
The dispersion relation for a phonon in the case of diatomic materials in 3D form. **a** The predominant routes of phonon decay start from their origin center as LO phonons and progressively turn into lower energy acoustic phonons that are responsible for the dissipation of heat. **b** The four types of decay mechanisms are depicted as Klemens, Ridely, Vallee–Bogani, and Barman–Sirvastava. The figures are taken with permission from ref. ^13^

The dominant way of heat dissipation, observed in polar compounds such as GaAs, occurs through Fröhlich interactions or Fröhlich scattering, which is governed by long-range coulombic potential. The initial rate of the intervalley carrier–LO phonon scattering is given by the following equation

4 $$\frac{1}{{\tau _{e - {\mathrm{LO}}}}} = \frac{{e^2\omega \sqrt {m_{{\mathrm{ef}}}} }}{{4\pi \varepsilon _0\hbar \sqrt 2 }}\left[ {\frac{1}{{\varepsilon _\infty }} - \frac{1}{{\varepsilon _s}}} \right] - \frac{1}{{\sqrt E }}\left[ {\ln \frac{{\sqrt {E + \sqrt {E - \hbar \omega } } }}{{\sqrt {E - \sqrt {E - \hbar \omega } } }}} \right]\left[ {\frac{{\eta _{BE\left( {\omega ,T} \right)}}}{{\eta _{BE\left( {\omega ,T} \right) + 1}}}} \right]$$

where *E* represents the energy above the CBM, *m*_ef_ is the electron effective mass, $\eta _{BE\left( {\omega ,T} \right)} = \frac{1}{{e^{\frac{{\hbar \omega }}{{k_BT}} - 1}}}$ is the phononic equilibrium population at *T* (temperature) with phonon frequency *ω*, $\varepsilon _\infty \,and\,\varepsilon _s$ are the dielectric constants at low- and high-frequency (static) constants. The same equation can be written for the hole cooling process in the valence band. Thus, the energy relaxation rate is given by $\left\langle\frac{{{\mathrm{d}}E}}{{{\mathrm{d}}_t}}\right\rangle = \frac{{\hbar \omega }}{{\tau _{e - {\mathrm{LO}}}}}$ by neglecting the re-absorption of the phonons. The cooling of HC (electrons) continue by LO phonon emission until their energy is less than the energy of an LO phonon ($\hbar \omega _{{\mathrm{LO}}}$) above the CBM. Interaction of the hot electrons with the acoustic phonons also takes place, but mainly around the Brillouin zone center, and exhibits negligible energy exchange while primarily imparting the momentum equilibration. LO phonons exhibit low thermal conductivity and the heat dissipation is mainly due to acoustic phonons. The lattice anharmonicity is responsible for the further decay of optical phonons to acoustic phonons. At temperatures <1000 K, there are four prominent decay mechanisms found in various materials^14^. In the cubic crystalline structure of zinc blend or diamond, three decays mechanisms are found: (i) Klemens decay mechanism, (ii) Ridley decay mechanism, and (iii) a decay mechanism through Valle´e–Bogani channel, and (iv) the fourth decay mechanism called Barman–Srivastava (Fig. 6b), is found in hexagonal materials (e.g., wurtzite structure). Generally, the electron–LO phonon scattering occurs in the time range of 1 ps, followed by the emission through acoustic phonons. Hence, the charge carriers become available for transfer to ESC or recombination (radiative or nonradiative).

## HC in organic–inorganic halide perovskites

**Fig. 7:**
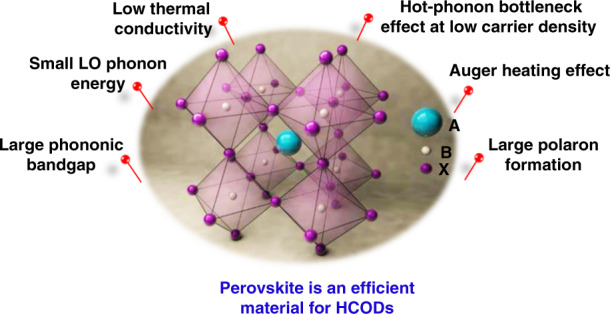
Organic-inorganic halide perovskite emerge as promising material with outstanding hot carrier properties. The figure shows various properties favorable for designing efficient hot carrier optoelectronic devices (HCODs)

Organic–inorganic metal halide perovskites have attained a top position in photovoltaic research due to their extraordinary optoelectronic properties, facile synthesis routes, and convenient device fabrication processes. Among these properties is slow HC cooling^28,31^, which is paramount for the new generation of HCCs to surpass the S–Q limit^32^. Recent experimental results revealed that the bulk lead halide perovskite materials showed slower HC dynamics at an excess excitation energy of 1.4 eV (∼0.4 ps)^33^ as compared to most of the other inorganic semiconductors (∼0.1 ps)^34^. The various intrinsic properties of perovskite materials that are responsible for prolonging the HC thermalization time are shown in Fig. 7.

### Polaron formation in perovskites and its size effect on HC cooling

**Fig. 8:**
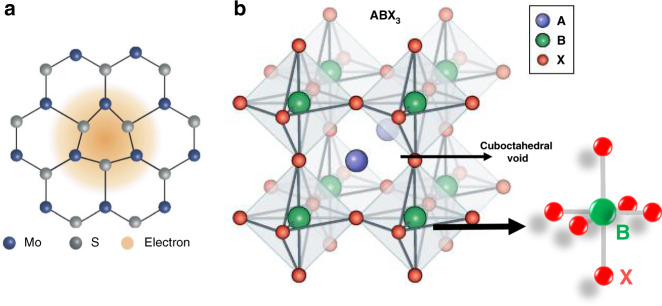
The schematic illustration of crystal structure in MoS_2_ and lead halide perovskite. (**a**) polaron formation in MoS_2_ and (**b**) the crystalline structure of lead halide perovskite (MAPbX_3_) that favors large polaron formation. The figures are taken with permission from (**a**) 38 and (**b**) 39 and modified. The figures are taken with permission from (**a**) ref. ^147^ and (**b**) ref. ^148^ and modified

The polaron concept was first introduced by Landau, and its self-energy and effective mass were investigated later^35^. A polaron originates from the interaction of a charge (electron or hole) with its surrounding atoms. Typically, in a polar semiconductor or an ionic crystal, the conducting charge carrier, together with its self-induced polarization cloud, forms a quasi-particle known as polaron (Fig. 8a)^36^. As compared to the bare charge particle, physically, a polaron is characterized by its newly modified properties, such as the polaron binding energy *E*_p_, its effective mass m^*^, and its response to the externally applied electric and magnetic field (e.g., DC mobility and optical absorption coefficient). These very peculiar properties lead to a strong change in the electrical and thermal properties of the materials^37^.

Two distinct types of polarons, small polaron and large polaron, are found in two- or three-dimensional systems. The type of polaron formation depends on the electron–lattice interaction, which is of primary importance. A large polaron forms due to the long-range Coulombic interactions between a charge carrier and a solid ion. Competing effects then determine the radius of the large polaron. By contrast, a small polaron can form when a short-range electron–lattice interaction, such as the deformation-potential interaction, is dominant^38^. Specifically, due to the excess charge in a deformable solid, the electron–phonon coupling becomes sufficiently strong so that a self-trapped polaron formation is found. For a strong electron–phonon coupling in highly polar and ionic crystalline solids, two driving forces are responsible for the polaron formations, namely (i) the long-range Coulombic potential (*V*^LR^) between the ionic lattice and the excess electron (screen hole), and (ii) the short-range deformation potential (*V*^SR^) due to the variation in local bonding by the excess charge. *V*^LR^ is given by the following Eq. (5)

5 $$V^{{\mathrm{LR}}}(r) = - \left[ {\frac{1}{{\varepsilon _{r( \propto )}}} - \frac{1}{{\varepsilon _{r(0)}}}} \right]\frac{{e^2}}{{\left| r \right|\varepsilon _0}}$$

where *r* is the vector distance between an ionic site and electron, $\varepsilon _0$ is the permittivity in vacuums, $\varepsilon _{r( \propto )}$ and $\varepsilon _{r(0)}$ are the high-frequency and static dielectric constants, respectively and $e$ is the electron charge. In the case of Si or GaAs, the two dielectric constants are the same ($\varepsilon _{r(0)} \approx \varepsilon _{r( \propto )}$), hence the value of $V^{{\mathrm{LR}}}$ is negligible. However, in ionic solids, the value of $\varepsilon _{r(0)}$ is twice that of $\varepsilon _{r( \propto )}$, thus making the long-range Coulombic potential significant. If *V*^LR^ is higher than *V*^SR^, the size of the polarization cloud (coherent length, *L*_coh_) is larger than the unit cell dimension (*L*_coh_ > a). This results in the formation of a large polaron. In the case of small polaron formation, *V*^SR^ is higher than *V*^LR^ (*L*_coh_ < a)^39^. The electronic and optical properties of large and small polarons are significantly different. The transport in large polarons is coherent with their high mobility (*µ* > 1 cm^2^ V^−1^ s^−1^) and the transport resembles the coherent transport of a free charge carrier in a conductor. However, mobility decreases with a decrease in temperature ($\frac{{\partial \mu }}{{\partial T}} < 0$). In contrast, the transport in small polarons is incoherent with far lower mobility (*µ* ≪ 1 cm^2^ V^−1^ s^−1^) and increases with the increasing temperature ($\frac{{\partial \mu }}{{\partial T}} > 0$)^38^. Lu et al. theoretically explained the polaron formation and their dynamics in organic semiconductors^40^. The large polaron is also defined as the shallow bound state that arises from the Coulombic interaction between the electron and the polarizable lattice. Similarly, small polarons are strongly localized wave functions within a chemical bond.

The unique crystal structure of organic–inorganic lead halide perovskite is a hybrid framework-like structure composed of two interpenetrating structures, (i) a sublattice of inorganic Pb halide corner-shared octahedra (PbX_6_)^4−^ to form a 3D crystalline network with $PbX_3^ -$ stoichiometry, and (ii) a sublattice of an organic cation (A^+^), commonly CH_3_NH_3_^+^, to fill the cubo-octahedral void between the inorganic sublattice and to balance the charge (Fig. 8b). The peculiar nature of this hybrid structure lies in such a way that both valence and conduction bands for charge transport are formed by the inorganic sublattice. This structure gives rise to two main properties, namely dynamic disorder and intrinsic softness. The dynamic disorder is reflected by various phenomena, such as the anharmonicity and broadening in far-infrared (far-IR) and low-frequency Raman spectra, the disorder in nuclear magnetic resonance (NMR) and X-ray or neutron scattering, and liquid-like responses in dielectric function or fs-ps responses^41,42^. Intrinsic softness is shown by lower Young’s moduli (i.e., ten times lower than Si and GaAs)^42^. Thus, the organic sublattice serves as charge screening and charge localization by modulating the electrostatic landscape experienced by the charge carriers^39^.

The dipole nature of organic molecule (CH_3_NH_3_^+^) introduces polar potential and ferroelectricity in halide perovskite structures. The fast rotations of this organic molecule and also the soft inorganic sublattice affect the carrier transport by electron–phonon coupling^43,44^. With a relatively strong electron–phonon coupling, large polaron formation is observed, which develops pseudo-free dressed carriers that screened from other free carriers and defects to avoid recombination and trapping. This results in a long lifetime and diffusion length^39^. These large polarons spread over a hundred unit cells due to their weak long-range Coulombic interaction and small lattice distortion of LO phonons. The HC longer lifetime (>100 ps) observed at low excitation density (<10^18^ cm^−3^) might be attributed to the screening of these large polarons. At high excitation density (>10^18^ cm^−3^), the overlapping of the polarons is responsible for the “phonon bottleneck” effect.

The formation of a small polaron is usually facilitated by the presence of lattice defects that initiate the charge trapping process with a negative impact on the transport of charge carriers as well as on the overall device efficiency^39,45^. Therefore, the effects of polarons on the charge transport properties need to be revealed in order to synthesize and design semiconductors for efficient optoelectronic devices.

### HC dynamics in perovskites at high- and low-carrier densities

Before analyzing in more detail the effects of high- and low-carrier density on HC dynamics, it is worth discussing how to calculate their density and the power or energy loss rate of HC. Spectroscopic techniques, such as TRPL and TA, are typically used to study the HC dynamics in nanocrystals (NCs) and thin films. Specifically, these techniques allow researchers to study the momentum and kinetic energy of the excited states along with their transient inhabitants (i.e., HC) and also provide essential information about the electronic states and the electronic structures of photoactive materials.

In a typical spectroscopic experiment, a laser beam of a selected power (typically in microwatt scale) is focused onto a small spot on the sample to excite it. The pump fluence (*F*) can be calculated as ${{F}} = {{EA}}^{ - 1}$, where *E* is energy and *A* is the spot size in cm^2^. Energy can be calculated as ${{{\mathrm{E}}}} = {{{\mathrm{power}}}} \times {{{\mathrm{time}}}}(f^{ - 1})$ where time is the repetition time of the used pulse, i.e., the inverse of frequency ($f^{ - 1}$). The carrier’s density calculations (n_0_ cm^−3^) in perovskite films has been reported by Dursun et al.^46^ as

6 $$n_0 = j \times \alpha$$

where *j* is the laser pump fluence and its unit is µJ cm^−2^ (equivalent to the number of photons/cm^2^) and can be calculated as *E* = n‧h‧c/λ), and $\alpha$ is the absorption coefficient at the excitation wavelength. The power loss rate of HC can be calculated by fitting the TA spectra and using the following equation^47^

7 $$P = \frac{{{\mathrm{d}}U_{\mathrm{C}}}}{{{\mathrm{d}}t}} = \frac{d}{{{\mathrm{d}}t}}\left( {\frac{3}{2}K_BT_{\mathrm{C}}} \right)$$

where *K*_*B*_ is Boltzmann constant, *T*_C_ and *T*_L_ are the temperatures of HC and lattice, respectively. Different cooling dynamics in perovskite films and NCs have been observed at different carrier densities or pump fluences. The rather complex interplay of HC cooling dynamics depends not only on the intrinsic properties but also on several additional factors, such as (i) the excitation energy, i.e., pump energy, with typically higher excess energy leading to a longer carrier’s lifetime, (ii) the initial HC density, where the higher density usually follows a longer cooling time trajectory, and (iii) HC energy loss rate, which is typically lower at lower HC temperature^13^.

**Fig. 9:**
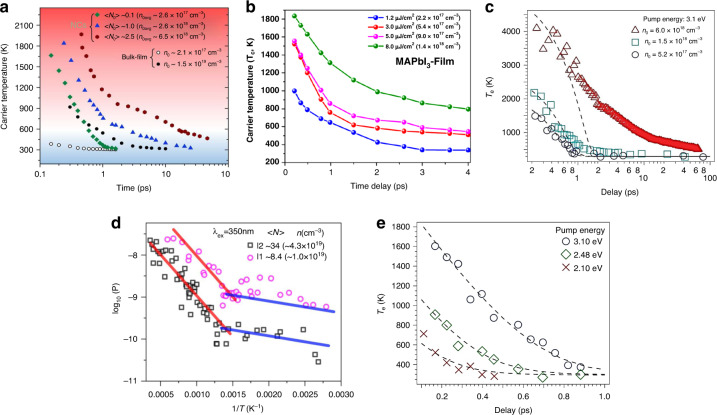
Effect of charge carrier densities and excitation energy on HC cooling dynamics. **a** The temperature of HC versus the delay time for MAPbBr3 NCs and bulk film. **b**, **c** represent the influence of various excitation densities on HC temperature on perovskite film, where higher initial carrier density corresponds to delay in cooling time. **d** Power loss in CsPbBr_3_ NCs as a function of inverse temperature, where the red line and blue line correspond to HC temperature higher than lattice LO phonons and the equilibrium established between both entities, respectively. **e** Slow cooling of organic lead halide perovskite in which the influence of various pump energies on HC cooling is observed. Higher energy corresponds to slow cooling. The figures are taken with permission from (**a**) ref. ^46^, (**c**) ref. ^50^ and (**d**, **e**) ref. ^47^

At low pump fluence where the carrier concentration is <10^18^ cm^−3^, the cooling dynamics can be explained by Eq. (7). However, at high pump fluence (carrier concentration after the excitation >10^18^ cm^−3^), the cooling trend deviates from the equation due to the appearance of two phenomena, Auger recombination and phonon bottleneck effect that decrease the cooling rate. The effect of the initial carrier density and excitation energy on HC dynamics in perovskite bulk film and NCs has been extensively investigated by various researchers^13,46,47^. Li et al. studied the effect of pump fluence on carriers cooling in MAPbBr_3_ NCs and bulk film, where the higher initial temperature at high pump fluence, as well as the slower cooling rate, has been observed for NCs as compared to bulk film (Fig. 9a)^13^. The smaller *T*_C_ of the bulk film showed ultrafast cooling of HC and is beyond the temporal resolution of the TA equipment used in the experiment. Similarly, there is a rapid energy distribution through elastic scattering, i.e electron–hole scattering, at low pump fluence and electron–electron scattering at high pump fluence is responsible for lower-temperature regime and higher cooling rate^26^. Higher excitation fluence generates higher *T*_C_ and also a slower cooling rate is observed. The initial rapid cooling rate until 600‒700 K is attributed to the strong carrier–LO–phonon coupling, which establishes thermal equilibration between HC and LO phonons. Beyond 600 K a lower cooling rate to the band edges is observed due to thermal equilibrium between LO phonons and acoustic phonons^26,47^. Chen et al. studied the same cooling rate in organic halide perovskite NCs, which is attributed to carriers-phonon couplings at higher and lower-temperature regimes (Fig. 9d). The steep red line in Fig. 9d indicates that the carriers’ temperature is higher than LO phonon, while the blue line corresponds to the regime where there is equilibration between LO phonon and HC. The thermal energy between electron and LO phonon moves back and forth, and the process sometimes is known as phonon energy up-conversion^48^. The breakpoint between the two regimes occurs at 600 K. Beyond that temperature, LO phonons give away their energy to acoustic phonons and this usually takes place at a timescale of 3–5 ps^47^. In polar semiconductors, slow carrier cooling is observed at higher excitation intensity or higher carrier density. Thus, in these semiconductors, three to four orders of slow cooling is attributed to hot LO-phonon botttleneck^30,49^. At higher laser fluence, multiple exciton states are occupied and they lead to higher carrier density as well as higher temperature. The lifetime of HC in MAPbI_3_ film reaches ≈60 ps until 600 K, which is about two times higher than the lifetime at low pump fluence^50^. The HC cooling time regime in hybrid perovskites is 100 times longer than that of GaAs film and CdS microplates^26^.

Similarly, an increase in the excitation energy results in higher temperature, as shown in Fig. 9e. The excess excitation energy above the bandgap of the targeted material corresponds to *T*_C_ according to the energy relation *E* = 13/2*K*_*B*_*T*_C_, and it is equally distributed between both carriers (electron and holes). Lower excitation energy will result in a temperature decrease of the resultant HC. Finally, the HC cool down to the band edge and are available for collection or recombination (Fig. 2b). These results provide an insight into HC dynamics in high and low excitation densities, which can be beneficial for selecting the appropriate selective contacts for an efficient charge extraction.

### Modifications in chemical composition and their effect on HC dynamics

Extensive research efforts have been directed toward tailoring the HC dynamics by doping and using various chemical modifications, such as cation (A^+^) and halogen (X^−^) modifications in organic–inorganic lead halide perovskite materials^48,50–53^ Xing et al. studied the effect of HC cooling dynamics and extractions in Zn-doped CsPbI_2_Br at low photoexcitation level (10^17^ cm^−3^). The Zn-doped perovskite showed a reduced HC cooling rate, three times smaller than undoped perovskite, which is due to the improved film morphology and lower defect density. In addition, the nonadiabatic coupling between conduction bands and the introduction of relaxation channels due to Zn are mainly responsible for slow carrier cooling and fast extraction at interface^53^. Madjet et al. simulated the theoretical radiative relaxation dynamics of HC in lead halide perovskites and outlined how relaxation time depends on the halogen composition. The effect of cation modification on HC dynamics in perovskites was studied by using nonadiabatic molecular dynamic (NA–MD) combined with density functional theory. Due to the larger nonadiabatic coupling in the valence band states as compared to the coupling in the conduction band states, the hot holes relax faster than hot electrons, which is further attributed to the low recombination rates in the perovskite systems^52^.

**Fig. 10:**
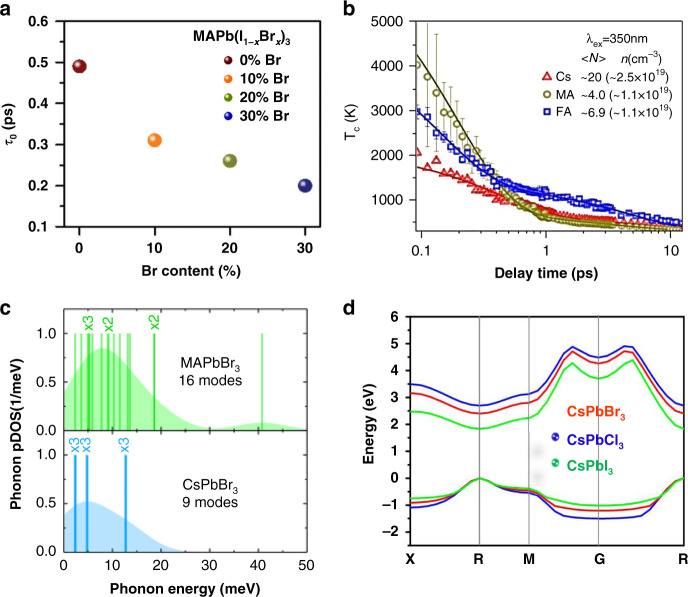
Effect of chemical modifications on HC relaxation dynamics in organic–inorganic lead halide perovskite. **a** The rapid thermalization of HC with increasing Br concentration can be seen, which indicates the strong electron–phonon coupling that in fact reduces the phonon bottleneck effect. **b** The dependence of HC cooling on cation nature where FA and MA cations are more prone to charge carrier–phonon coupling compared to Cs counterpart. **c** Energy and density of states of phonons in MAPbBr_3_ (16 modes) and CsPbBr_3_ (9 modes) and **d** the band structure of all inorganic perovskite (CsPbX_3_, X = Cl, Br, I) crystal showing the highest valence band (VB1) and lowest conduction band (CB1) and their energy of states. The figures are taken with the permission from (**a**) ref. ^54^, (**b**) ref. ^47^, (**c**) ref. ^58^, and (**d**) ref. ^51^

Cation modification is a simple and easy method to prolong the HC lifetime in lead halide perovskite materials. In a similar study, an increase in chlorine (Cl) concentration leads to slower HC relaxation dynamics which is due to nonadiabatic electronic coupling that arises from charge-state localization around the Cl atom. In addition, in a work by Talbert et al. the effect of bromide content in MAPbI_3-x_Br_x_ on the excited states and HC dynamics was probed^54^. With an increase in Br concentration, there is not much effect on auger recombination, but the rapid thermalization of HC was observed due to enhanced electron–phonon coupling. A strong hot-phonon bottleneck effect has been observed for pure MAPbI_3_, which indicates a long HC lifetime, while an increase in Br substitution in crystal lattice suppresses the phonon bottleneck effect. Figure 10a shows the substantial rapid thermalization with an increase in Br contents in mixed-halide perovskites.

In recent years, A-site cation engineering of the ABX_3_ perovskite structure, with A being e.g., formamidinium (FA), methylammonium (MA), and/or inorganic (e.g., Cs) cation, has been a hot topic for the study of HC dynamics, and many interesting fundamentals have been revealed by ultrafast spectroscopy techniques^47,48,51,55^. Due to defect tolerance, nanostructure, quantum confinement, and excellent charge transport properties, the low-dimensional halide perovksite [e.g., quantum dots (QDs), nanocrystals (NCs), nanowires (NWs), and nanorods (NRs)] have gained much research attention. Recently, long-range exciton diffusion length (>10 µm) has been observed in MAPbBr_3_ NC film that has shown a superior exciton carrier mobility (10 cm^2^ V^−1^ s^−1^) which is much higher than 3D perovskite film^56^. Chen et al. related the HC relaxation dynamics to the cation engineering in lead halide perovskite (MAPbBr_3_) NCs and observed longer HC cooling time for inorganic (Cs) cation-based perovskite compared to the other organic counterparts (MA and FA) (Fig. 10b)^47^. The fast motion of the organic cations (MA and FA) induces stronger electron–phonon interaction and coupling as compared to Cs^57^. As a result, enhanced HC cooling is achieved for organic cation-based perovskites. Among the three selected cations (i.e., MA, FA, and Cs), FA showed the fastest relaxation dynamics. This was ascribed to its strongest interaction with the PbX framework as compared to the other two cations. The intrinsic thermal conductivity of the material has also an impact on HC lifetime, and longer lifetimes are displayed for materials with lower thermal conductivity. Hopper et al. studied the HC relaxation of different lead halide perovskite systems by fs TA spectroscopy, where he studied the effect of cation and halide composition (e.g., FAPbI_3_, FAPbBr_3_, MAPbI_3_, MAPbBr_3_, and CsPbBr_3_) on the HC temperature and relaxation dynamics^58^. The inorganic cation (Cs) showed the longest HC relaxation and prolonged cooling time compared to other configurations, which was attributed to a smaller specific heat capacity^59,60^ and fewer optical phonon modes in Cs-based halide perovskites (9 optical phonon modes for CsPbBr_3_ and 16 for MAPbBr_3_) as shown in Fig. 10c. The valence band (VB) and conduction band (CB) are mostly occupied by lead and the incorporation of halogen can affect the bandgap as well as density of states (DOS) of organic–inorganic perovskite. In CsPbX_3_ when X sequentially altered from Cl to I, there is a change from 3p to 5p, observed in valence orbital that corresponds to a decrease in perovskite bandgap. The effect of halide modification on the band structure in Cs-based halide perovskites can be seen in Fig. 10d, where the lowest bandgap (1.83 eV) is achieved with the iodide substitution. Intrinsic thermal conductivity in perovskite materials is relatively lower as compared to other semiconductors, which has a positive impact on HC dynamics by prolonging their lifetime^61^. The single and polycrystalline MAPbI_3_ perovskites were also studied, displaying ultralow thermal conductivities of 0.5 WmK^−1^ and 0.3 WmK^−1^, respectively. The lower phononic group velocities and anharmonicity were responsible for such a slow thermal conductivity^62^.

These results will guide future works on how the phonon modes in different materials affect the relaxation mechanism and dynamics of HC relaxation. HC dynamics are mostly cationic dependant where organic cation (FA and MA)-based perovskite shows faster thermalization as compared to inorganic (Cs) perovskite. The HC cooling times of various perovskite compositions are summarized in Table 1.

**Table 1:** HC cooling dynamics in various perovskite materials

Composition	HC (HC) cooling time (fs)	Technique applied for measurement	Ref.
CsPbBr_1.5_Cl_1.5_	471	Femtosecond transient absorption spectroscopy [f-(TA)]	^51^
CsPbBr_2_Cl_1_	450		
CsPbBr_3_	765		
CsPbBr_1.5_I_1.5_	591		
CsPbBr_1_I_2_	760		
CH_3_NH_3_PbI_3_	700 fs (electrons), 600 fs (holes)	[f-(TA)]	^87^
CsPbBr_3_ NCs	400	[f-(TA)]	^47^
MAPbBr_3_NCs	200		
FAPbBr_3_ NCs	150		
FAPbI_3_, FAPbBr_3_, MAPbI_3_, MAPbBr_3_, and CsPbBr_3_.	100–900	Pump-push-probe (PPP) ultrafast spectroscopy	^58^
MAPbBr_3_ single crystals	150 ± 30 ps	TRPL	^146^
FAPbI_3_ and MAPbI_3_ NCs	~30 ps	f-TA	^50^

### HC cooling and multiple exciton generation (MEG)

HC typically possess an amount of energy equal to the difference between the bandgap of the photoactive material and the incident photon energy. However, if this excess photon energy exceeds a certain threshold limit (two times *E*_g_), the MEG process takes place, which yields two or more excitons^9,63^. MEG has been proposed as a promising and feasible method for capturing the energy of HC and boosting the light conversion beyond the S–Q limit^7,64^. MEG can be promoted in semiconductor NCs compared to their bulk counterparts^7^. Fortunately, the slow HC cooling in perovskites makes them highly suitable for MEG, which has led to discoveries of the underlying physics of the MEG phenomena^9,65,66^.

After the excitation of HC, the carrier thermalization and cooling processes depend on the properties of the HC and the band structure of the photoabsorber. In optoelectronic applications, the carrier scattering rates determine the fundamental limits of carrier transport and electronic coherence.

### Quasi-ballistic HC diffusion

By combining TA with spatial microscopy, researchers such as Sung et al.^67,68^ and Guo et al.^69^ showed that HC can diffuse extremely fast compared to the cooled carriers. While the cooled carriers may require a few hundred ps to few nanoseconds to travel across a 500 nm thick perovskite layer, the HC can travel at near a ballistic speed with a traveling distance of 150 nm within the first 20 fs^68^, and 600 nm overall before cooling down^69^. This implies that the excess energy of the hot electrons can act like kinetic energy. By improving the perovskite quality via reduction and passivation of traps, this ballistic transport can be further enhanced^67^. These results suggest much better chances for functional HC as the HC extraction is otherwise limited to the carriers close to the extraction interface.

## Working principals of HC solar cells

**Fig. 11:**
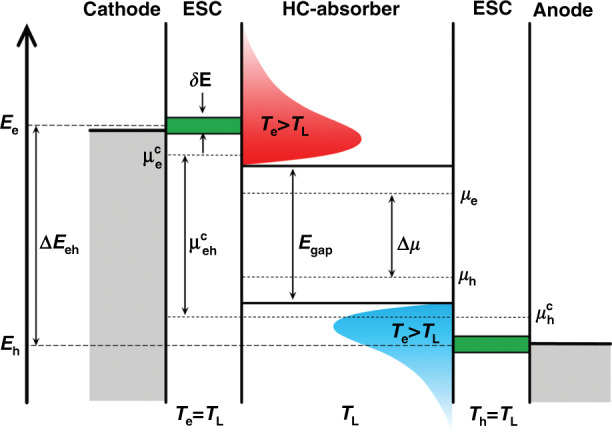
The energy diagram of a HC-absorber, ESCs, and the respective electrodes. $\mu _e^c$ and $\mu _h^c$ are the chemical potential energies for hot electrons and holes, respectively, and $\mu _{eh}^c$ is their separation. The figure is taken with permission from ref. ^14^

To handle the major PV loss mechanisms due to the fast thermalization and cooling of HC, it is important to understand the working principle of these novel types of devices, namely HCSCs. To implement the ideal and theoretical model of HC to a working device, two parameters are of crucial importance; (i) long cooling time and (ii) ultrafast and efficient extraction of HC before their cooling. This innovative HC solar cell design was proposed since 1982 by various eminent researchers^4,5,15,70^ and is equivalent to a conventional solar cell, where an absorber is sandwiched between the two very thin carrier extraction layers followed by external electrodes, as shown in Fig. 11.

The basic operation of a HC solar cell is to utilize the temperature gradient established between the HC (*T*_C_) and the lattice (*T*_L_) to get higher voltages than are otherwise obtainable in a conventional PV cell^4^. ESCs extract HC with specific energies (*E*_e_ for hot electrons and *E*_p_ for hot holes) with an energy window of δ*E* from the absorber. The bandwidth of the energy selective window (ESW) has to be narrow, less than *k*_*B*_*T*_L_, in order to prevent the HC from relaxing to the lower states and cooling down isentropically to *T*_L_. The open-circuit voltage of the device is given by Eq. (4),

8 $$eV_{{\mathrm{OC}}} = \mu _{{\mathrm{eh}}}^c = {\Delta}E_{{\mathrm{eh}}}\left( {1 - \frac{{T_{\mathrm{L}}}}{{T_{{\mathrm{eh}}}}}} \right) + {\Delta}\mu \frac{{T_{\mathrm{L}}}}{{T_{{\mathrm{eh}}}}}$$

where ∆*E*_eh_ is the energy separation of the ESCs. However, in a conventional solar cell, the equation reduces to $eV_{{\mathrm{OC}}} = {\Delta}\mu$ as the HC relax to *T*_L_. In conventional PV devices, there is much emphasis on particle extraction rather than energy extraction^25^.

### Physics of the energy-selective contacts for the HC extraction

**Fig. 12:**
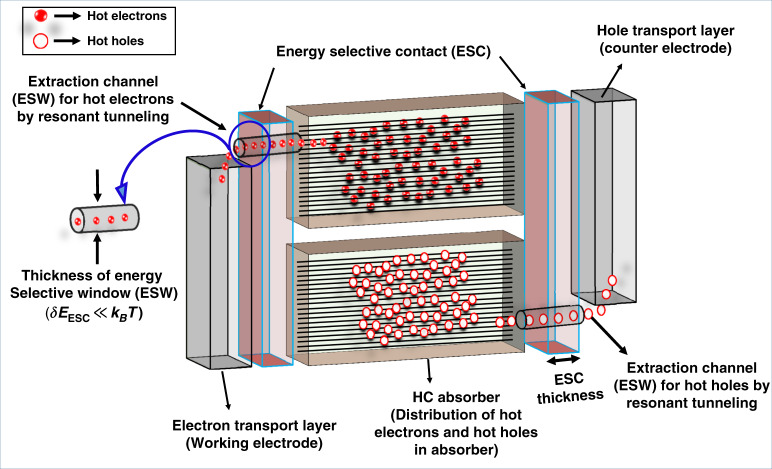
The schematics showing HC solar cell. Energy selective window (ESW) with thickness δE << K_B_T serve as channel for selective and efficient extraction of hot charge carriers through photoabsorber

The selection of an ideal and effective ESC is difficult as it acts as a tunneling barrier with a resonant energy level of width ${\Delta}E_{{\mathrm{ESC}}}$ ($\delta {{E}}_{{{{\mathrm{ESC}}}}} < < k_BT$) allowing only a narrow range of charges to tunnel through it. However, the HC beyond the aforementioned energy have a high probability to reflect back into the absorber. The isentropic extraction of the HC through the ESC prevents the mixing of HC with colder ones during their transfer and thus minimizes the increase in entropy. Figure 12 illustrates this process.

### Hot carriers in other semiconductors and energy-selective contacts

**Fig. 13:**
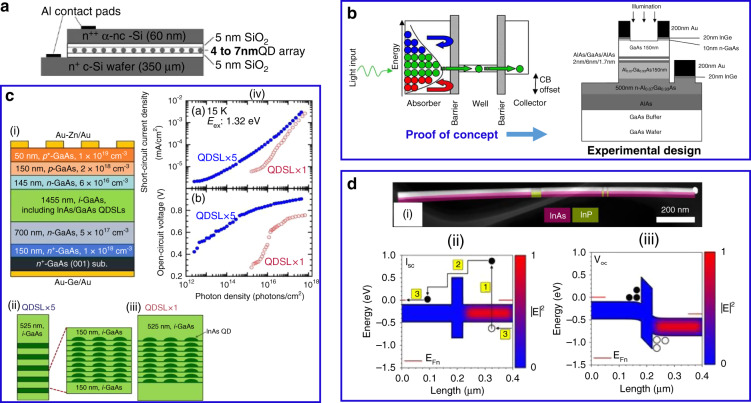
The schematics and device architecture for HC extraction through energy-selective contacts in various semiconductors. **a** 2–4 nm Si quantum dots are sandwiched between SiO_2_ layers. **b** Hot electrons resonant tunneling concept where the hot electrons with resonant tunneling energy easily cross the barrier while those with non-resonant energy reflect back to the absorber. The concept was experimentally proven with GaAs as the absorber and AlGaAs as the hot-electron collector. **c** InAs/GaAs layered based quantum dot solar cells: (i) the schematics of stacking InAs/GaAs quantum dot layers, (ii and iii) the InAs QD layers are embedded in a GaAs matrix, (iv) stacking multiple QD layers enhances both current and voltage of the device, and **d** a single nanowire-based HC solar cell: (i) the device cross-section taken with the scanning electron microscope (SEM), (ii) energy band diagram showing three steps in the current generation (I_SC_) process as electron–hole pair photo-generation, their diffusion and charge transfer and, (iii) the energy band diagram under *V*_OC_ condition. The figures are taken with permission from (**a**) ref. ^73^, (**b**) ref. ^78^, (**c**) ref. ^71^, and (**d**) ref. ^82^

The theoretical concept of HC extraction from a wide-bandgap material with narrow conduction and valence band through an ESC membrane was introduced by Würfel^15^. According to that concept, 85% efficiency can be achieved via the exclusion of the electron–phonon scattering mechanism in the absorber material. Various nanostructures such as quantum dots (QDs), quantum wells (QWs), and nanowires (NWs) were suggested as appropriate candidates for ESCs^8,16,71,72^. Later, the experimental evidence of HC extraction was achieved by using 4–7 nm thick Si QDs array which was sandwiched between two SiO_2_ layers (with the thickness of 5 nm each) (Fig. 13a)^73–75^. This array acts as a medium with double-barrier resonant tunneling for HC extraction. Similarly, Su et al. simulated HC by using QDs and QWs as the ESC where the former showed significantly improved efficiency and performance^8^, and the experimental evidence by the same author supported his simulation data^76^. The parameter of conductance of ESC using QDs and QWs was neglected previously. Since then it has been shown that a resonant tunneling diode (RTD) with QDs and QWs may be negatively affected by both high or too low conductance^77^. However, plenty of experimental research has been done to prove the concept of resonant tunneling using QDs and QWs^28,72^. A similar demonstration of a proof of concept of hot-electron extraction has been presented based on resonant tunneling from a narrow bandgap GaAs (absorber) to a wider bandgap AlGaAs (collector) through a double-barrier QWs of AlAs/GaAs structure^78,79^. The device temperature ranged from 93 to 213 K where the electron–phonon interaction was negligible. However, promising results can be achieved by optimizing the barrier thickness, selection of appropriate absorber with slow carrier-cooling rates, and reducing dimensionality from 3D to 2D. The schematics and proof of concept are shown in Fig. 13b. Recently, in 2019, Dimmock et al. showed an enhanced HC extraction through resonant tunneling using semiconductor QWs as ESC^80^. A few nanometer thick metallic layer (chromium) was used as the photoabsorber on GaAs substrate and by altering the thickness of the metallic film, the absorption of incident light was enhanced that resulted in the generation of HC. The two different mechanisms, tunnelling and thermionic emission of hot charge extraction from metal to semiconductor were studied. All these materials discussed above are relatively wide-bandgap materials (i.e *E*_*g*_ > 1 eV). However, theoretical maximum power conversion efficiency for HC can be achieved with low-bandgap materials (i.e *E*_*g*_ < 0.5 eV)^8,24^ and single nanowires with unidirectional morphology^81^. In this context, InP was used as the ESC to harvest photogenerated hot electrons from a single NW of InAs (*E*_*g*_ = 0.39 eV)^82^. The author has claimed an increase in short circuit current by adopting certain strategies and modifications in the NW such as variation in diameter, surface passivation, and modification in the ESC that showed an enhancement of the current collection.

The schematics of a single NW solar cell are presented in Fig. 13d. Very recently in 2019, Harada et al. demonstrated the successful extraction of HC in an InAs/GaAs QDs superlattice solar cell by employing successive layers of QD lattices, which increased the photo-absorption, current, and voltage of the device compared to a single QD lattice^71^. Table 2 represents various types of ESCs used for HC extraction in conventional semiconductors and organic-inorganic halide perovskites.

**Table 2:** Energy-selective contacts employed in various HC

Type of energy-selective contacts (ESCs)	Photoabsorber	Temperature of the measurement	Equipment used for the measurements	Ref.
4–7 nm array of Si QDs	SiO_2_	RT	Optically excited I–V measurement	^73^
2–7 nm array of Si QDs	SiO_2_	RT	Optically excited I–V measurement	^74^
QDs and QWs	Materials selected with bandgap, *E*_g_ = 1 eV	Simulated at 300 K	Theoretical model	^8^
InGaAs QWs	GaAs	10 K for lattice >35 K HC	I–V and PL	^72^
AlAs/GaAs Double barrier QWs	GaAs	93–213 K for lattice 477 K for electron	I–V	^78^
AlAs/GaAs Double barrier MQWs	GaAs	Lattice temperature 140 K	TDPL and TRPL	^79^
InP NWs	InAs	Device temperature 6–300 K	I–V	^82^
AlGaAs QWs	Chromium metallic absorber	80 K to RT	I–V	^80^
InP Thin Fim	PbSe	RT	Double beam optical setup	^28^
QDs	InAs/GaAs	15 K	I–V setup	^71^
B-Phen	CH_3_NH_3_PbBr_3_	RT	f -TA	^13^
Graphene	CH_3_NH_3_PbI_3_	RT	f-TA	^83^
B-Phen	CH_3_NH_3_PbI_3_	RT	PPP spectroscopy	^84^
C_60_	CH_3_NH_3_PbI_3_	RT	f-TA	^83^

*RT* room temperature

**Fig. 14:**
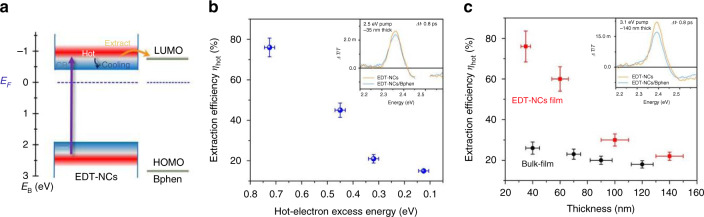
HC extraction by using extremely thin absorber (B-Phen) as energy- selective contact. **a** The schematics of HC generation and their extraction with an illustration of the flat energy band alignment of B-Phen absorber and perovskite NCs. **b** the dependence of the pump energy on HC extraction using a 35 nm thin B-Phen layer in which hot electrons with higher excitation energy can be extracted efficiently. **c** The HC extraction efficiency versus the B-Phen layer thickness for both the perovskite bulk film and NCs at 3.1 eV photoexctation. Higher extraction of HC was observed for NCs. The figures are taken with permission from ref. ^13^

Although perovskites have demonstrated slow HC cooling and are considered a game-changer for the future of HCSCs, they require a proper understanding of suitable and efficient ESCs that can strongly couple their energy bands with those of the perovskite absorber^13,83–85^. Recently, researchers have employed various forms of carbon such as graphene and C_60_ as ESCs in perovskite solar cells, which have not only improved the current densities but also the stability of the devices. In 2017, Li et al. was the first to use a thin absorber layer of 4,7-diphenyl-1,10-phenanthroline (B-Phen) as the ESC with perovskite and studied the dynamics and extraction of HC in a system of MAPbBr_3_ NCs coated with a B-Phen, demonstrating efficient hot-electron extraction from the perovskite^13^. The B-Phen has high electron mobility, narrow electron bandwidth^13^ and a suitable molecular structure for strong coupling of its higher LUMO to the CBM of the perovskite (Fig. 14a). The HC extraction efficiency (*ƞ*_hot_) reaches up to 75% at low pump fluence but reduces to 58% when pump fluence is increased, which is attributed to the back electron transfer from the B-Phen into the perovskite. The perovskite NCs prevailed over their bulk counterpart when it came to the charge extraction efficiency (Fig. 14c). It has been observed that *ƞ*_hot_ reduces from 75 to 15% as the excess HC energy reduces from 0.7 to 0.1 eV, which is clear evidence that B-Phen has a strong coupling with the perovskite and only the HC with high temperature and energy have been extracted.

Recently, in 2019, Lim et al. revealed the formation of an interfacial junction between a perovskite absorber (MAPbI_3_) and B-Phen, which is responsible for the electron-back transfer to the absorber at higher pump fluence^84^. Instead of using conventional ultrafast spectroscopy, they deployed pump-push probe (PPP) spectroscopy, which has been used for probing the excited states by re-exciting the carrier population above the LUMO of the absorber with the push pulse after the initial pump. PPP has the ability to circumvent the various complexities that arise because of the multiband excitation and density-dependent multiparticle effects to probe the lifetime of the excited state^84^. At 3.1 eV pump pulse, the HC are not fully extracted by B-Phen, and thus no quenching is seen (Fig. 14b). However, a 1.98 eV push probe successively transfers HC to B-Phen. The reason for this hindrance in a complete HC transfer might be caused by an interfacial Schottky barrier formed at the interface of the two materials (perovskite and B-Phen) and a single pump fluence of even 3.5 eV cannot overcome the barrier. Wang et al. reported a new device design (MAPbI_3_/Au/TiO_2_/FTO) in which photoexcited hot electrons were extracted under one sun steady-state illumination. Hot electrons traversed ballistically through Au film and transferred to Au-TiO_2_ interface^86^. These innovative findings pave the way for exploring efficient ESCs for highly efficient HCSCs.

Researchers have also observed an ultrafast charge transfer from a thick film of metal halide perovskites to the electron- and hole-accepting layers (ETL and HTL, respectively) by monitoring the transient absorption spectra of perovskite-ETL/HTL systems in the near-infrared (NIR) region, where the transient spectra of a pristine perovskite film differed greatly from the spectra of perovskite-ETL/HTL systems^87,88^. This difference in the signal was attributed to the ultrafast charge transfer in fs timescale while the charge carriers are still hot. However, these results were later challenged by the findings showing that the NIR TA spectra of perovskite films primarily originate from the photoinduced change in reflectance and thin-film interference, not from a change in absorption^89^. The interference of the pristine film and the resulting TA spectra are modified by the presence of ETL or HTL. Thus, no charge extraction takes place and perceived HC transfer is nothing but likely a mischaracterization of the TA signal. Adding an ETL/HTL would therefore modify the thin-film interference of the pristine film and the resulting TA spectra even if no charge extraction took place, and the perceived HC transfer was likely a mischaracterization of the signal.

## Plasmonic HC and their applications in other optoelectronic devices

**Fig. 15:**
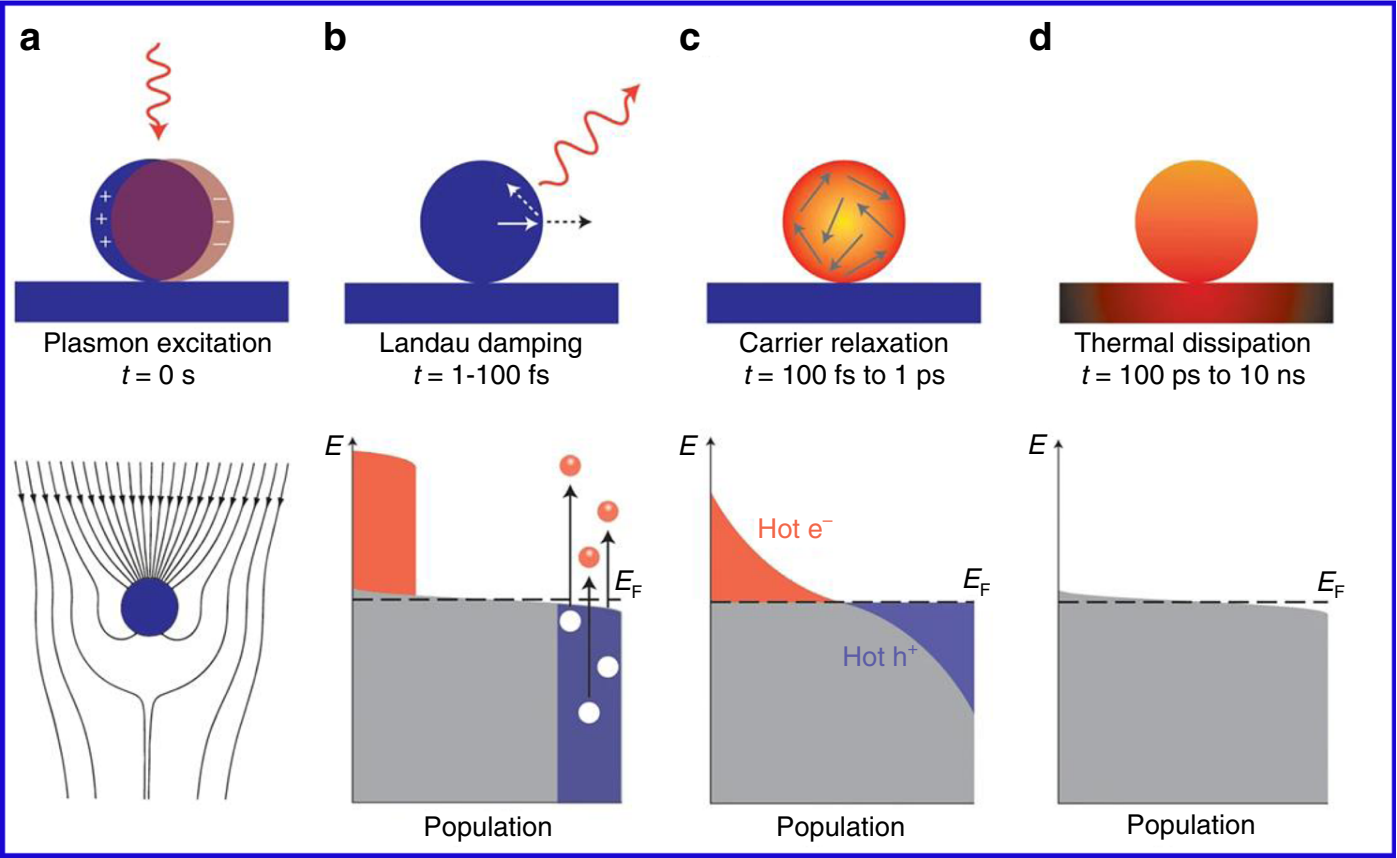
A schematic representation of the plasmon excitation and dephasing in a metal nanoparticle and the characteristic timescale. **a** Photoexcitation of the local surface plasmons and enhanced light absorption. **b** HC generation through Landau damping (1‒100 fs) and their subsequent decay through photon re-emission or by carrier multiplication. **c** HC redistribute their energy quickly (100 fs‒1 ps) through carrier–carrier scattering processes. **d** Finally, heat is transferred out of the metallic structure to the surroundings at a relatively long timescale (100 ps–1 ns). Electronic states are represented by gray areas, the hot holes and hot electrons are presented in orange and purple colors, respectively. The figures are taken with permission from ref. ^149^

For centuries, scientists have been fascinated by the interaction between incident light and matter. In properly designed metallic nanostructures (typically Au, Ag, and Cu), if the frequency of incident light matches with that of the free electrons, the collective oscillations of the electrons are triggered in a confined region^90^. This oscillation reaches its maximum amplitude at a specific wavelength known as the localized surface plasmon resonance (LSPR) (Fig. 15a) and the surface plasmon polaritons (SPPs). The collective oscillation is called a plasmon. The LSPR is damped either radiatively through re-emission of a photon or by the nonradiative decay, also named as Landau damping, by the generation of HC within the plasmonic nanostructures^91,92^. The generation and decay processes of HC in a plasmonic nanostructure are displayed in Fig. 15a–d. The Landau damping is a quantum mechanical phenomenon in which the LSPR quantum is transferred to a single-electron–hole pair excitation at an ultrafast timescale (1‒100 fs) (Fig. 15b). Finally, HC redistribute their energy and the heat is transferred to the surrounding environment.

**Fig. 16:**
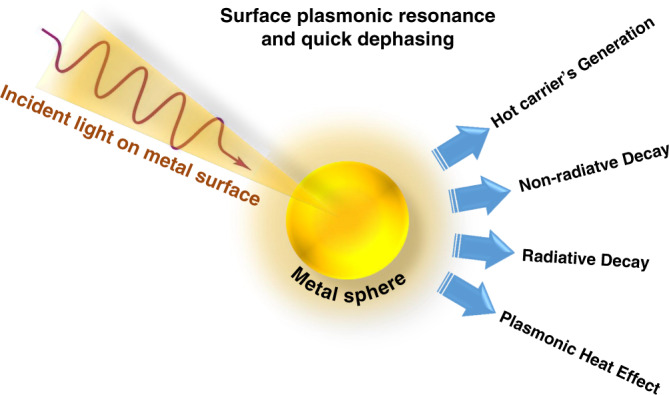
Representation of the dephasing of plasmons and their energy dissipation mechanism. Energy released through four mechanisms: HC generation, nonradiative decay, radioactive decay, and plasmonic heating effect

Thus, typically a plasmon dephases quickly and the energy decays via four different routes, namely (i) HC generation, (ii) near-field electromagnetic field enhancement (non-radiatively), (iii) far-field light scattering (radiatively), and (iv) plasmonic heat effects (Fig. 16)^93,94^. The ratio of each of these routes depends on the morphology as well as the surrounding medium of the plasmonic nanostructure.

In recent years, extensive research has been done to exploit the novel aspects of plasmonic HC in carefully synthesized and designed metallic nanostructures to effectively exploit the decay energy into various outstanding applications^91,93,95^. Some potential applications include enhanced infrared absorption from a near-field enhancement effect known as the surface-enhanced Raman scattering effect (SERS), enhanced light trapping far-field radiation effect, cancer therapy with plasmons, water splitting and other photoconversion routes for photochemistry, photovoltaics, photodetection, and sensing^96,97^ applications. Various applications of HC are explained in detail for better understanding and development of future optoelectronic devices.

### HC-mediated photochemical reactions

**Fig. 17:**
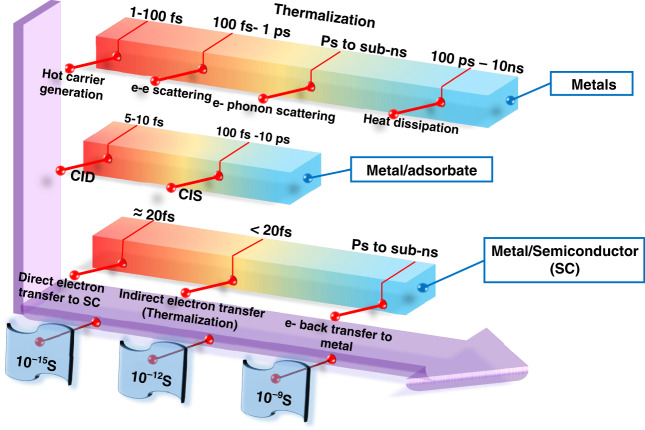
The timescale of the plasmon-induced HC generation, their transfer, and thermalization processes in the presence of an adsorbate of semiconductor. Note that SC stands for semiconductor, e stands for electron and CID & CIS refer to the chemical interface damping and the chemical interface scattering, respectively. The idea of the figure has been taken from ref. ^102^, modified and redrawn

During the photoexcitation of metallic nanoparticles, the oscillation of the plasmonic electrons results in the confinement of photon energy at the metallic surface over a longer period of time compared to the unconfined photons that travel at speed of light, therefore tremendous accumulation of photon intensity and highly energetic hot electrons are created at the surface of the nanoparticle^98,99^. When adsorbate molecules or semiconductors are directly attached to a metal nanostructure, hot electrons can be captured and extracted to the adsorbates or semiconductors before thermalization. The effect of light illumination on the distribution of hot electrons and their self-scattering (electron–electron scattering) and interaction with phonons have been theoretically studied as well as experimentally explored for plasmonic nanostructures^19^. Due to the rapid relaxation through self-scattering, only a very small portion of the plasmonic hot electrons can overcome the interfacial energy barrier for the indirect charge transfer process. The hot-electron injection process is in competition with the electron–electron scattering process. Besides the back transfer of electrons taking place at the interface, the self-scattering and the scattering with phonons are some of the basic reasons that the indirect transfer process shows a very low efficiency (typically < 2%). There are two possible ways for plasmons to decay in the metal/adsorbate system; one is a direct pathway (the generated hot electrons are transferred to the LUMO of the attached molecule) and the other is an indirect pathway (the hot electrons are generated in the LUMO of the attached molecule and leave a hot hole behind in the metal)^100^. The direct pathway of the plasmon decay is also termed as the chemical interface damping (CID)^101^. Apart from the thermalization of HC through their conventional scattering process, there is another way for their thermalization in metals called chemical interface scattering (CIS), in which a hot charge carrier upon transferring to the adsorbate moiety leaves a portion of its energy behind and transfers back to the metal again. This portion of the energy vibrationally excites the adsorbate, transforms it into a hot adsorbate^102^ and turns the hot adsorbate into a transient energy reservoir that prolongs the HC lifetime from fs to ps, thus making them perfectly available for a chemical reaction^102^. The various timescales for the hot-electron generation, as well as their relevant transfer to the adsorbate or semiconductor, are illustrated in Fig. 17 (see refs. ^100–103^).

**Fig. 18:**
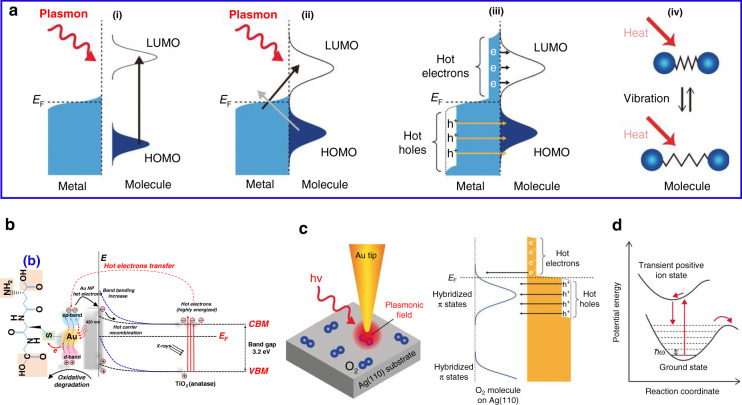
Schematics of the surface plasmon-mediated chemical reactions with the HC before thermalization. **a** The excitation mechanisms in plasmonic-induced reactions representing (i) direct intramolecular excitation, (ii) excitation between metal and adsorbate, (iii) HC transfer to adsorbate, and (iv) local heat generation through the decay of localized surface plasmons that cause vibrational excitation. **b** The interfacial chemistry and charge transfer mechanism of thiolated Au/TiO_2_ excited through X-rays and visible light radiations. **c** Illustration of the experimental setup and plasmon-induced disassociation of a single O_2_ molecule when it adsorbed on Ag surface and **d** HC transferred to hybridized π^*^ states of O_2_ molecule and potential energy curve for O_2_ molecular dissociation. The Figures are taken with permission from (**a**, **c**, **d**) ref. ^106^ and (**b**) ref. ^107^

Similarly, HC generation and their vital role in photocatalytic behaviors in metal, metal–semiconductor, and hybrid nanostructures have been widely accepted^104^. In a typical catalytic reaction, the HC either directly or indirectly transfer to the adsorbate molecule or semiconductor. Indirect transfer suffers large energy losses due to the HC self-scattering and scattering with phonons. However, a direct transfer required a suitable and empty hybridized orbital of an adsorbate or semiconductor with strong interaction^105^. The excitation mechanism presented by plasmon chemistry is shown in Fig. 18^106^. In direct intramolecular excitation, when the adsorbate molecule attached to the metal surface, is excited resonantly with LSP, there is a direct transition occurs between frontier electronic states of adsorbate (Fig. 18a-i). In direct excitation, the charge transfers between metal and adsorbate states (Fig. 18a-ii) thus HC transfer from metal to adsorbate molecule through inelastic tunnelling (Fig. 18a-iii). Local heating generates through decay of LSP involve in the reduction of the activation barrier and vibrational excitation of adsorbate molecules (Fig. 18a-iv).

A strong motivation towards the replacement of fossils fuels, photoreduction of available stocks of H_2_O (water splitting) and CO_2_ have been a matter of interest for a long time. As compared to water splitting, the photoreduction of CO_2_ is a more challenging and complex process. However, plasmonic HC can facilitate the photoreduction of CO_2_ attached at the surface of a metal oxide. Recently, Chu et al. performed a theoretical simulation of the photoexcited electron-induced CO_2_ reduction at the surface of a rutile TiO_2_^105^. Due to the excitation of specific vibrational modes, the CO_2_ molecule can trap hot electrons and dissociate to form CO within 30–40 fs. However, the transient life of the CO_2_ molecule is very short (10‒15 fs), which is due to the strong hybridization of the CO_2_ orbitals with the TiO_2_ (rutile 110 surface) electronic band. However, the time limit for the photoreduction may vary for other metal oxides. Similarly, HC transfer at metal/metal oxide interfacial heterojunction has been thoroughly investigated due to the widespread applications in phototherapy, photocatalysis, sensing, and removal of hazardous pollutants. Au/TiO_2_ is one such example: the hot electrons in Au can initiate the photoreduction while the hot holes are left in the valence band of TiO_2_, which were used in oxidative degradation of thiol-ligand (L-glutathione capping ligands) attached to the Au surface (Fig. 18b)^107^. The reaction mechanism of the photo-redox reaction, where the plasmon generates and initiates the redox cycle, is still unclear. Because these reactions generate HC, strong electric fields, and heat by excitation and decay processes, it is unclear which factor is controlling the mechanism.

Very recently, chemists have studied the governing factors responsible for the plasmon-induced chemical reactions and explored “why shining light on silver nanoparticles causes oxygen molecules, attached to their surfaces to break off”^106^. Christopher et al. investigated the HC-mediated plasmon-induced oxidation of various chemical moieties chemisorbed on the Ag surface^108^. Similarly, others have studied the same mechanism responsible for the plasmon-induced activation or disassociation of an O_2_ molecule^109,110^. However, Seemala et al. found that the disassociation of the O_2_ molecule attached to Ag is due to the interaction of the localized surface plasmon (LSP) and the molecule rather than HC involved^111^. Therefore, to clarify the fundamental mechanism of this reaction, Kazuma et al. studied a single-molecule chemisorbed on the Ag surface by using a scanning tunneling microscope (STM) combined with a light illumination source^106^. The experimental setup is illustrated in Fig. 18c in which the Au tip was positioned on the target molecule and excites through LSP. Using a combination of theoretical calculations and experimental results, they revealed that although there is a contribution of hot electrons, however, hot holes transfer is the dominant mechanism of the O_2_ disassociation^108^. The coupled structure that forms due to the molecule–metal chemical interaction is the source of the HC transfer to the antibonding molecular orbital of a strongly hybridized oxygen. The schematics for a plasmonic-induced HC-mediated O_2_ reaction mechanism with a potential energy surface has been illustrated in Fig. 18d in which the transfer of the hot holes to the strongly hybridized π^*^ states of the adsorbed O_2_ molecule is detailed. These simulations and experimental studies proved that the HC transfer and the degree of hybridization between the metal state and the metal oxide’s surface are the principal mechanisms of the molecular excitation by LSPs.

**Fig. 19:**
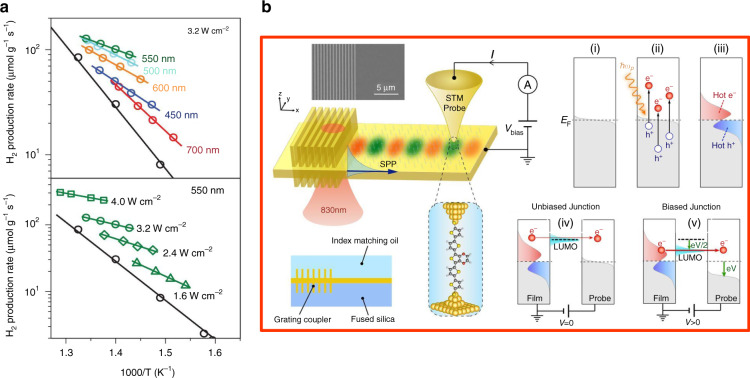
The effect of HC in photoinduced plasmonic chemical reactions. **a** The quantification analysis of reaction mechanism with and without HC generation in terms of light intensity and wavelength in hydrogen production and **b** the schematics of the experiment where a single-molecule junction was established between a grounded nanodevice and the STM probe, alongside the HC generation and their distribution. The figures are taken with permission from (**a**) ref. ^112^ and (**b**) ref. ^113^

In plasmonic photocatalysis, the generation of HC and their importance in photochemical reactions through metal–semiconductor hybrid materials have been extensively investigated^104,112–114^. Zhou et al. studied the role of HC in plasmonic photocatalysis of ammonia (NH_3_) by using plasmonic antenna reactor (AR) photocatalyst of copper ruthenium (Cu-Ru) surface alloy that composed of Cu nanoparticles (NPs) and Ru reactor sites^112^. The conventional thermal decomposition of NH_3_ for the production of H_2_ requires high temperature because of the high thermal activation energy (*E*_a_) of 1–2 eV to achieve the turnover frequency (1 NH_3_ molecule/active metal site/second)^115^. However, by using AR, the reaction rate was higher as compared to the Cu and Ru NPs alone, and an enhancement of the turnover frequency (>15%) was also achieved. The decomposition of ammonia upon excitation has been investigated as a function of excitation wavelength and intensity^112^ (Fig. 19a). It was observed that without any external heating (high temperature), the plasmonic surface with the AR photocatalyst gave the highest decomposition rate of NH_3_ at an illumination of 9.6 Wcm^−2^.

The energy distribution of the plasmon-generated HC in the metallic nanostructures is highly important for the design and development of highly active plasmon-driven applications^116^.

Reddy et al. investigated the steady-state energy distribution of the HC with STM by creating a single molecular junction (quaterthiophene, tetracyanoethylene or 3,4 thylenedioxythiophene) between an Au thin film (6–13 nm) and the Au tip of the STM (Fig. 19b)^113^. By employing various biasing voltages, the current–voltage characteristics were elucidated with and without plasmonic excitations, which allowed them to calculate the energy distribution of the HC. According to their findings, the film thickness, the molecular type, and the distance between the STP tip and Au film are the crucial factors that contribute towards the HC current and their energy distribution above the Fermi level. An increase in the thickness of the Au film (13 nm) reduced the HC generation by 43% as compared to a thinner Au film (6 nm).

Overall, the fundamental understanding of HC generation, energy distribution, transfer, and energy decay mechanism are crucially required for the development of highly efficient and sensitive plasmonic photocatalytic applications, and there is still much left to know about the HC generation through the surface plasmonic polariton. Various important topics are still under investigation, including for example the energy losses due to the vibrations of the adsorbate molecule, the interfacial energy loss by the electron–electron scattering, and the contribution of both thermal and plasmonic HC on the plasmonic catalysis.

### HC in transistors and photodetectors

The development of transistors operating in the high-frequency region (terahertz, THz) is the key requirement for future imaging, sensing, and communication applications such as the next-generation autonomous vehicles and wireless communication systems^117,118^. Currently, for such applications, high-electron-mobility transistors are designed by using III‒IV semiconductor heterostructures in which lateral charge transport is governed by field-effect modulation^119,120^. However, there is a hindrance in further improving these devices due to the technical and physical issues related to the lateral scaling of the channel. Hence, as an alternative to lateral field-effect transistors, hot-electron transistors are a suitable vertical design composed of an ultrathin base layer, which facilitates transverse ballistic transport of hot electrons. The typical device architecture consists of three terminals (emitter, base, and collector) and, under a base-to-emitter polarization condition, the hot electrons are injected from the emitter to the base region. If the base thickness is shorter than the mean free path, the hot electrons can transit through the base region without any energy loss (ballistic transport)^121,122^. These THz transistors rely on a channel material that can accommodate high carrier densities suitable for low-contact resistance^123,124^.

**Fig. 20:**
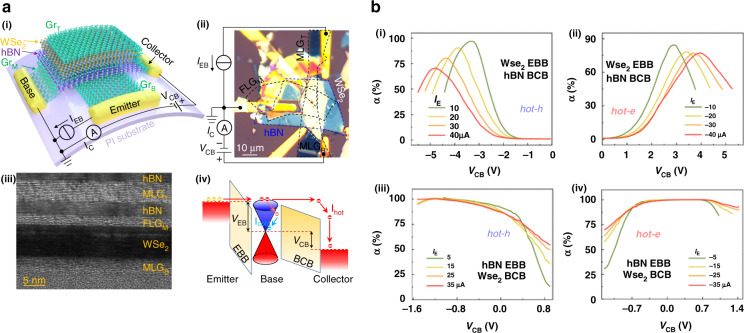
The device architecture of a 2D Van der Wall hot-electron transistor and the related graphs for the HC collection efficiencies. **a** The HET device design on a flexible substrate showing the electrical connections, along with the optical image, TEM image of the real device and the energy band diagram representing the HC energetics and **b** the collection efficiencies of HC under various bias conditions. The figures are taken with permission from ref. ^121^

Giannazzo et al. used graphene in a junction with an Al_x_Ga_1-x_N/GaN heterostructure and evaluated the hot-electron injection efficiency from the AlGaN/GaN into the graphene base region^122^. They achieved a high on-state current density (*J*_c_) of 1 Acm^−2^. Similarly, Liu et al. recently observed ambipolar HC transport for the first time in a newly proposed device architecture of a HET transistor for the efficient collection of HC^121^. The typical device structure consists of an extra thin (~1 nm) layer by layer stacking of graphene, hexagonal boron nitride and Tungsten diselenide. They achieved nearly lossless ambipolar transport of both hot holes and hot electrons through an extra thin base (Fig. 20a, b) and almost 100% collection efficiency. This very high collection efficiency is due to several reasons, such as (1) long HC lifetime and less carrier–phonon interactions in the graphene, (2) high-energy and high-injection levels reduce the chances of HC backscattering at the collector’s barrier, and (3) efficient charge transfer due to a clean interface and strong adhesion between the different stacked layers.

The device architecture with the thin graphene base not only enhanced the efficiency limit of the HET but also provided a platform for the fundamental understanding of HC dynamics in these high-frequency devices. However, in graphene-based heterostructures, the scattering of HC has a negative impact on the charge transfer properties, especially scattering due to the field injection of hot holes. The reason for the HC scattering even in an ultrashort (10 nm) gate is still a topic to be addressed^125^. Perovskite materials with longer quasi-ballistic transport, HC lifetime, and easy fabrication process are a strong hope for the development of hot-electron transistors.

**Fig. 21:**
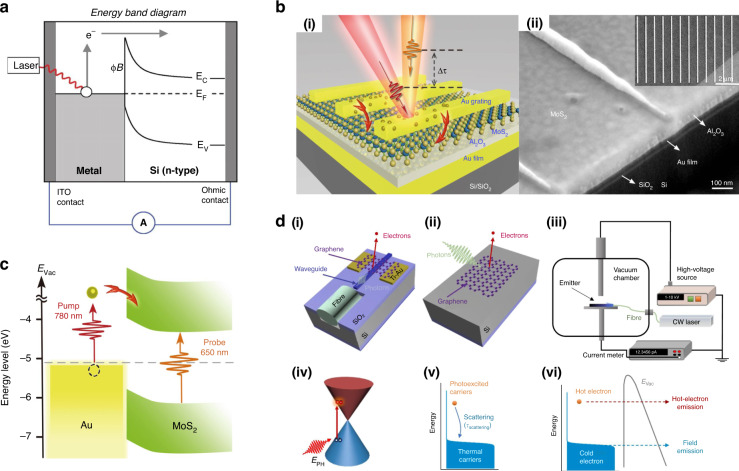
In plasmonic photodectors various nanostructures (metals and graphene) when embedded in a semiconductor, serve as optical antenna and facilitate the efficient HC generation and transfer to the semiconductor. **a** The energy band diagram of an optical antenna diode showing the photoexcitation and injection of hot electrons into the Si over the Shottky barrier (∅_*B*_), thus contributing to the photocurrent generation. **b**–i: The schematic of the sandwiched heterostructure device with an Au grating/MoS_2_/Al_2_O_3_/Au/Si, and **b**-ii a cross-section image of the MIM structure of a real device. **c** A diagram showing the band alignment of the Au grating and MoS_2_ monolayer where the plasmonic hot electrons were excited by a 780 nm pump laser, and by crossing the Schottky barrier they are injected to MoS_2_ monolayer, where the induced variation in the filled states is monitored by a 650 nm probe pulse. **d** The detailed schematics of a complete device model and experimental setup of a graphene photoemitter: (i–iii) the mechanisms of the hot-electron generation and scattering, the hot-electron emission prior to cooling, the field emission of thermal electrons, and (iv–vi) the continuous wave-laser photoemission by the waveguide mechanism of an integrated monolayer of graphene. The figures are taken with permission from (**a**) ref. ^17^, (**b**, **c**) ref. ^140^, and (**d**) ref. ^131^

In optical communication, high-speed photodetectors are an important component that converts the optical photons into electrical signals^126^. Specifically, photodetectors are able to detect photoelectrons from a metal surface over a Schottky barrier or an oxide tunnel barrier^127^. The presence of a Schottky barrier (∅_*B*_) limits the motion of HC through metal/metal oxide interface and the efficient injection of hot electrons into the semiconductor remains a major challenge. Besides, HC must have enough energy to overcome this barrier, and their momentum should be in the perpendicular direction to the interface. Thus, both energy and momentum are the two primary requirements, as they are the main causes of the low extraction efficiency of HC^128^. The embedding of optical nanoantennas within the semiconductor facilitates the HC extraction throughout the surface of nanoantenna and circumvents this issue. Knight et al. fabricated a rectangular array of Au nanorods (optical antenna) on a silicon substrate, which generates hot electrons via plasmon decay^17^. The energy band diagram of the optical antenna diode is presented in Fig. 21a. A detectable photocurrent was produced via the absorption of light by the metal (Au) nanorods and the generation of hot electrons that emit over the Schottky barrier. These plasmonic nanoantennas have the capability of detecting light below the bandgap of the semiconductor (in this case, Si) even without a voltage bias. Although multiple antenna arrays could make an efficient on-chip spectrometer, the current device can only absorb 0.01% of the photons. A further enhancement in the efficiency could be achieved by optimization of the materials and structural morphology.

Similarly, graphene has been explored as an extraordinary plasmonic material due to its long lifetime and exceptional optical confinement with comparatively low losses^129^. In presence of static electric potential, the electrons in graphene can reflect, refract, and interfere in similar manner like electromagnetic waves in dielectrics. Photon-like behavior of charge carriers in graphene 2D morphology makes this material an excellent candidate for exploitation of electron optics^130^. The waveguide photoemission by using graphene monolayer has been experimentally tested in the work of Rezaeifar et al., where the probability of the photoemission was enhanced before the thermalization of the hot electrons^131^. At room temperature, the HC effect in graphene is prominent because of the relatively large electron–electron relaxation rate in comparison to its electron–phonon relaxation rate, which enables the fast and sensitive THz detection through photothermoelectric effect^132–135^. However, the important challenge in graphene is to increase its absorption. Plasmonic photodetection may greatly enhance the spectral range and sensitivity of the currently available light-harvesting devices. Due to their extraordinary performance and upscaling ability graphene-based mid-infrared and THz detectors are recently much focused^136,137^. Cai et al. introduced graphene-based THz detectors that worked at room temperature^138,139^. Fabrication of large-area arrays of graphene microribbons that were oriented perpendicularly on SiC substrate greatly enhanced the absorption efficiency in THz range.

HC generation through plasmonic oscillation, especially by the interaction of LSP and the SPP, is a complex phenomenon. In a recent study, Shan et al. proposed a new device design to observe the generation of HC through a strong coupling of LSP and SPP^140^. The proposed new metal-insulator-metal (MIM) heterostructure (Fig. 21b), in which molybdenum selenide (MoS_2_) serves as a heterojunction, showed strong coupling between the LSP and SPP that synergize to produce plasmonic HC. The electrons are excited by a 760-nm pump laser, and by crossing the Schottky barrier these electrons are injected into the MoS_2_ layer where they are monitored by a probe pulse of 650 nm (Fig. 21c). In contrast to the LSP that release their energy radiatively by emitting a photon, the SPP decay non-radiatively, leading to high photon-carrier conversion efficiency. However, in practice, these carriers generated by the SPP decay have not enough energy to cross the potential barrier between the metal and the semiconductor which results in low output yield. Thus, instead of transformation into useful electrical energy, the photon absorbed by the SPP are mostly converted to charge carriers which exhausted as heat^140^. The properties of LSP and SPP can be synergize to produce plasmonic HC. In this coherent energy exchange mechanism, the photons radiated by the relaxation of the LSP are reabsorbed by the SPP and thus usable in HC generation. In the work by Shan et al.^140^, the ultrafast (≈40 fs) transfer of hot electrons from the Au grating to the MoS_2_ monolayer achieved an external quantum yield of 1.65%.

### The outstanding performance of perovskites in plasmonic devices

The heterostructure that consists of metal and semiconductor NPs are employed in plasmon-induced hot-electron devices. At the metal–semiconductor interface, the efficiency of plasmon-hot-electron conversion strongly determines the performance of these devices. replacement of a conventional chalcogenide semiconductor (II–IV, e.g., CdS and CdSe) with a perovskite (CsPbBr_3_) has substantially improved the efficiency of the plasmon-hot-electron conversion at the Ag–CsPbBr_3_ interface^141^. The quantum efficiency (energy resonant transfer) of the device was enhanced up to ≈15%, which is attributed to the fast (<100 fs) transfer of HC at the interface. As compared to other conventional semiconductors with similar bandgaps, the large absorption cross-section of CsPbBr_3_ NCs and the large and strong oscillation strength of the intraband transition are responsible for efficient plasmon-hot-electron conversion at the metal/perovskite interface^142^. Similarly, compared to the ballistic transport length (85 nm) of GaAs grown through molecular beam epitaxy^143^, perovskite (MAPbI_3_) can achieve a much longer length (>200 nm) despite of their economical low-temperature and solution-processable fabrication^67,69^. Hence, for designing the ultrathin base layer of the HET, perovskites can be suitable candidates due to the longer ballistic transport length. The enhanced efficiency (~50 ± 18%) of the plasmonic-induced HET and the long-living charge-separated energy states prove a promising potential in the employment of the nano-heterostructures with the metal–perovskite semiconductor to further improve the performance of the hot-carrier optoelectronic devices. Recently, Gu et al. fabricated a MAPbI_3_–Au-based photodetector in which the generation of HC is promoted through the enhanced field of LSPR^144^. Due to the enhanced electromagnetic field and the suppressed electron–hole recombination, the MAPbI_3_–Au-based photodetector showed a very short response time and a large photocurrent.

Although the noble metals (Au, Ag, etc.) are well-known for their plasmonic effects, their direct contact with the perovskite may induce degradation, which is a serious limitation on the device lifetime of the noble metal–perovskite hybrid structures^144^. However, metal nitrides (TiN and ZrN) have also been successfully incorporated as plasmonic nanostructures. Mohsen et al. recently developed a model using COMSOL multiphysics simulation, which showed that by decorating perovskite (MASnI_3_) with core–shell nanostructures of ZRN/SiO_2_, an unprecedented increase in efficiency up to 20% could be achieved^145^. In this simulated architecture, the core–shell structure acts as a nanoantenna with an effective coupling of the SPR to the underlying perovskite layer, substantially increasing the optical absorption of the perovskite.

The timescale of the plasmonic HC functionality ranges from fs to ps (<10 ps) before the thermal equilibration. However, the timescales for various applications such as photochemical reactions, efficient HC collection in transistors, sensing, and detection, are much longer (fs‒ps) than the lifetime of the plasmonic HC. Therefore, their fast and efficient extraction from the photoabsorber or the plasmonic material needs to be addressed in the future. Nonetheless, the highly efficient plasmon-hot-electron conversion can be applied to further improve the emerging technology of HC optoelectronic devices based on perovskite semiconductors.

## Outlook for HC in future optoelectronic devices

The fundamental understanding of HC generation, thermalization, and cooling phenomena has not only explored the basic science of higher molecular states but also opened new pathways for the use of HC in a variety of innovative applications. For the successful collection of these highly energetic and unstable species, one needs to understand the energy decay processes of their self-scattering, scattering with phonons, and finally interactions with the lattice phonons. The ultrashort time span of these energy decay processes plays a vital role in determining the energy distribution of HC, their probability of transferring into nearby acceptor levels for efficient extraction, collection, or taking part in the chemical reaction, and finally designing a suitable device architecture. Although the increased confinement in a finite nanoparticle will definitely increase the HC lifetime due to the bottleneck effect, a highly efficient energy-selective contact is still vital for any final device. The factors that have a strong impact on the efficiency and can be tuned further to enhance the performance of HC optoelectronic devices are (i) HC relaxation time (*τ*_rel_), which has the largest impact on improving the efficiency. *τ*_rel_ should have a similar timescale as the radiative recombination, i.e 10‒100 ps, and the PCE could be increased up to 50 % if a timescale of 1 ns is achieved; (ii) heat dissipation characteristics, which can be considered in terms of the thermalization rate *Q*_th_. *Q*_th_ is recommended to be greater than 1 WK^−1^ cm^−2^ for a substantial HC contribution; (iii) HC equilibration time, and (iv) HC extraction time. Nevertheless, the effective and successful implementation of these concepts in a working device still remains a challenge for the future.

Perovskite materials have shown extraordinary performances in various optoelectronic devices, and they possess intrinsic extraordinary properties of slow cooling, reduced heat dissipation, efficient charge transport characteristics, and tuneable energy band alignment with other interfacial semiconductor or metallic nanostructures. Perovskites can be further explored for futuristic HC optoelectronic devices. As the HC lie in a pool of lattice phonons and the interaction with the phonons result in heat dissipation, one can think of introducing a suitable blocking mechanism at each stage of the thermal relaxation such as a thermal isolation between the phonons and the HC populations, the up-conversion of acoustic phonons to optical phonons and the reduction of the density of states (DOS) of the phonons. However, the effective temperature of the crystal lattice will be increased by employing such strategies, and how to modify the currently available perovskite materials for such a scenario, these are the questions for the future which need to be thoroughly addressed.

In addition to the importance of the photoexcited HC in high-efficiency photovoltaic applications, plasmonic HC are also expected to deliver new fundamental intuition into various dynamic processes at the interfaces and on the metallic surfaces, such as chemical reactions, desorption of molecules, and many more. The new strategies of embedding optical nanoantenna within semiconductor and the use of canonical metallic nanotips near the surface of semiconductors enable better HC generation and also employed for the enhancement of HC extraction at the interface. Their role in other applications such as photodetection, hot-electron transistors, lasing applications, and light-harvesting have been noteworthy. The efficiency of the new energy technologies, such as the hydrogen-based fuel cells, could be improved by the successful utilization of HC, which would enhance the chemical reactions. Besides the other energy loss mechanisms governing these conventional materials such as the HC self-scattering and scattering with phonons, there is another new route of their thermalization termed as the chemical interface damping also known as the ultrafast chemical interface scattering, which has been found in metal/adsorbate or metal–semiconductor hybrid systems. Although various landmark achievements have been secured, the enhancement in the device efficiency is generally weak in traditional hybrid nanostructures due to the low yield of HC along with their lower utilization rate. Similarly, the emergence of the layered perovskite materials and their ability to incorporate various cations, metals, and halides, could provide an ideal platform to investigate the generation of both photoexcited and plasmonic HC. The various coupling phenomena such as photon–phonon, photon–exciton, and electron–phonon will pave the way to explore the fundamental science and future technologies.

With the advances in device fabrication and characterization using ultrafast laser spectroscopy techniques, the HC optoelectronic applications are ripe for scientific breakthroughs. Insights into the HC dynamics in the higher energy states not only help conceptualizing new ground-breaking optoelectronic devices but also remain a hot fundamental research topic in the future.
